# Advances in Symbiotic Bioabsorbable Devices

**DOI:** 10.1002/advs.202410289

**Published:** 2025-01-23

**Authors:** Chang Zhu, Engui Wang, Zhou Li, Han Ouyang

**Affiliations:** ^1^ School of Nanoscience and Engineering School of Chemical Sciences University of Chinese Academy of Sciences Beijing 100049 China; ^2^ Beijing Institute of Nanoenergy and Nanosystems Chinese Academy of Sciences Beijing 101400 China

**Keywords:** biodegradable materials, biotherapeutics, symbiotic bioabsorbable devices

## Abstract

Symbiotic bioabsorbable devices are ideal for temporary treatment. This eliminates the boundaries between the device and organism and develops a symbiotic relationship by degrading nutrients that directly enter the cells, tissues, and body to avoid the hazards of device retention. Symbiotic bioresorbable electronics show great promise for sensing, diagnostics, therapy, and rehabilitation, as underpinned by innovations in materials, devices, and systems. This review focuses on recent advances in bioabsorbable devices. Innovation is focused on the material, device, and system levels. Significant advances in biomedical applications are reviewed, including integrated diagnostics, tissue repair, cardiac pacing, and neurostimulation. In addition to the material, device, and system issues, the challenges and trends in symbiotic bioresorbable electronics are discussed.

## Graphical abstract

This review focuses on recent advances in bioabsorbable devices. This review concludes innovation at the material, device, and system levels, the significant advances toward biomedical applications. This review discusses and highlights the challenges and trends in symbiotic bioresorbable electronics, and provides a new direction for the development of symbiotic bioabsorbable devices.

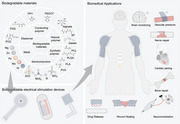

## 1 Introduction

Implantable medical equipment is a key development direction in modern medical technology.^[^ ^1^, ^2^, ^3^ ^]^ However, implantable electronic devices are limited by their high cost and risk of pain, infection, or immune reactions caused by the device itself.^[^ ^4^, ^5^, ^6^ ^]^ Symbiotic bioabsorbable devices has received widespread attention, due to their unique degradability that can be completely or partially dissolved,^[^ ^7^, ^8^, ^9^ ^]^ decomposed and absorbed by the human body through physical or chemical processes in the patient's body to eliminate the shortcomings of the implantable electronic devices themselves.^[^ ^10^ ^]^

Biodegradability refers to the ability to naturally degrade nutrients in the environment or within an organism.^[^ ^11^, ^12^ ^]^ Symbiotic bioabsorbable electronic devices and materials undergo degradation in the biological milieu through mechanisms such as material hydrolysis and metabolic processes within the body. The symbiotic relationship is developed by degradation into nutrients directly into cells, tissues, and the body to avoid equipment retention.^[^ ^13^ ^]^

**Figure 1:**
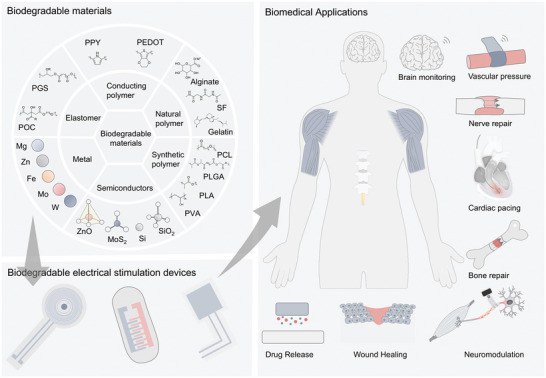
Symbiotic bioabsorbable materials, devices, and biomedical applications.

The inception of absorbable implantable electronic devices can be traced back to the integration of ultrathin transistors and logic gates into fibroin films, creating partially absorbable systems that incorporate semiconductor components.^[^ ^14^ ^]^ In this study, the materials used in absorbable electronic devices are summarized, emphasizing bioabsorbable sensors, energy storage devices, energy harvesting devices, and other devices, as well as the application of biotherapeutic systems such as integrated diagnosis, tissue repair, and cardiac pacing formed by special manufacturing and assembly methods. **Figure** **1** shows the direction of development of symbiotic biological devices. Recent advances in symbiotic bioabsorbable devices for biomedical therapy are also summarized.^[^ ^15^, ^16^, ^17^ ^]^

## 2 Materials for Symbiotic Bioabsorbable Devices

**Figure 2:**
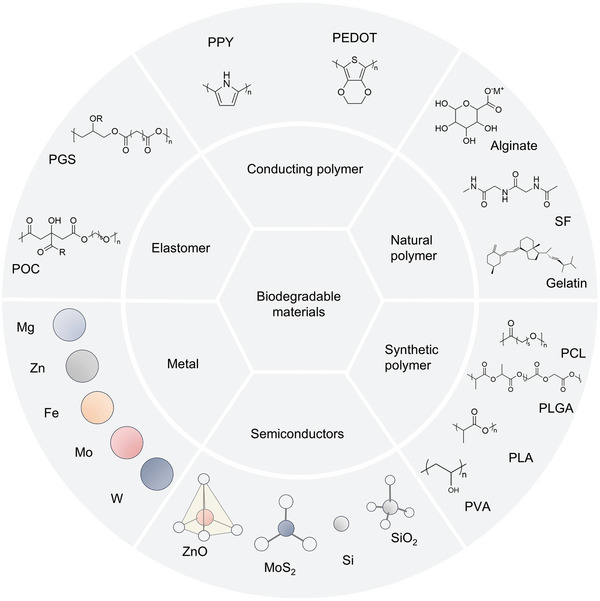
Symbiotic bioabsorbable materials.

**Figure 3:**
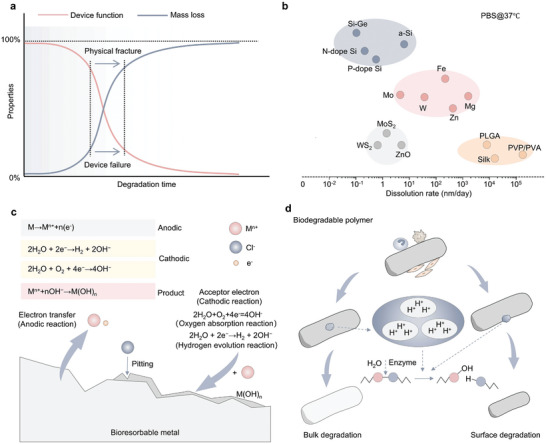
Schematic diagram of symbiotic bioabsorbable material degradation in vivo. a) The relationship between device function and mass loss. b) Degradation rate diagram of symbiotic bioabsorbable materials. c) Symbiotic bioabsorbable inorganic material degradation mechanism diagram. d) Symbiotic bioabsorbable polymer material degradation mechanism diagram.

Extensive research into bioabsorbable materials has led to the development of bioabsorbable electronic products. **Figure** **2** summarizes the types of symbiotic biomaterials. Bioabsorbable materials are mainly divided into two types: completely soluble in water/biological fluids and soluble byproducts.^[^ ^18^ ^]^ Symbiotic bioabsorbable systems typically comprise three components: functional materials, supporting substrates, and packaging materials.^[^ ^19^ ^]^ The degradation of the packaging material in the biological body or body fluids through the packaging layer into the functional material results in the degradation of closed functional components, ultimately leading to a reduction in the functional performance of the device until the final loss of the therapeutic effect (**Figure** **3**a). Therefore, the degradation kinetics and mechanisms of symbiotic biodegradable materials determine the functional life of these devices.

Symbiotic biopolymers are divided into naturally extracted polymers and synthetic polymers, which are mainly used in the supportive and packaging components of devices, the functional parts of which are more extensive. Functional organic materials and inorganic materials can be applied to the working part of the device.^[^ ^20^, ^21^, ^22^ ^]^ The preparation of the polymer part of symbiotic bioabsorbable electronic devices is relatively simple, achieved mainly by solution synthesis, whereas the synthesis process of inorganic materials is diversification, and different inorganic materials with different needs can be synthesized by different methods. There are relatively few reports on dissolution behavior of organic materials in vitro environment and organisms. This is because organic symbiotic biomaterials are mostly used in encapsulation layer, and their use in functional materials is less than that of inorganic materials. The degradation rates of symbiotic bioabsorbable materials are listed in **Table** **1**.

**Table 1:** Characteristics of bioresorbable materials for symbiotic bioabsorbable devices. (Results from degradation tests in PBS and temperature of 37 °C).

Materials	Type	Electrical property	Degradation time	Applications	Refs.
Mg	Metal	Conductors	1680 nm d^−1^	Electrodes, batteries	[23]
Mo	Metal	Conductors	0.7 nm d^−1^	Electrodes, supercapacitors	[24]
Zn	Metal	Conductors	168 nm d^−1^	Electrodes, batteries	[25]
Fe	Metal	Conductors	7 nm d^−1^	Electrodes, batteries	[24]
W	Metal	Conductors	20 nm d^−1^	electrodes	[24]
MoS_2_	Inorganic	Semiconductors	0.264 nm d^−1^	Electrodes, implants	[26]
ZnO	Inorganic	Semiconductors	96 nm d^−1^	Electrodes, implants	[27]
Si	Inorganic	Semiconductors	4.5 nm d^−1^	Electronics	[28]
Poly‐Si	Inorganic	Semiconductors	2.8 nm d^−1^	Implants	[29]
a‐Si	Inorganic	Semiconductors	4.1 nm d^−1^	Implants	[29]
SiO_2_	Inorganic	Semiconductors	8.2 nm d^−1^	Implants	[30]
Si_3_N_4_	Inorganic	Semiconductors	0.4 nm d^−1^	Implants	[31]
SF	Natural polymer	Insulators	100% (in protease solution) mass loss after 4 weeks	Scaffolds	[32]
Chitosan	Natural polymer	Insulators	90% mass loss after 1 d	Encapsulation	[33]
Alginate	Natural polymer	Insulators	20% mass loss after 40 d	Scaffolds	[34]
Candelilla wax	Natural polymer	Insulators	300 nm d^−1^	Encapsulation	[35]
Gelatin	Natural polymer	Insulators	68% mass after 15 d	Scaffolds	[36]
PLLA/PCL	Synthetic polymer	Insulators	100% mass after 100 d	Encapsulation	[37]
PLA	Synthetic polymer	Insulators	64%% mass loss after 12 months	Encapsulation	[38]
PGA	Synthetic polymer	Insulators	10% mass loss after 3 weeks	Scaffolds	[39]
PLGA(50:50)	Synthetic polymer	Insulators	80% mass loss after 8 weeks	Encapsulation	[40]
PCL	Synthetic polymer	Insulators	3% mass loss after 5 weeks	Encapsulation	[41]
PVA	Synthetic polymer	Insulators	90% mass loss after 90 d	Scaffolds	[42]
PHBV	Synthetic polymer	Insulators	4% mass loss after 15 weeks	Encapsulation	[43]
POMaC	Elastomer	Insulators	77% mass loss after 10 weeks	Scaffolds	[44]
PGS	Elastomer	Insulators	96% (enzymatic degradation) mass loss after 8h	Scaffolds	[45]
POC	Elastomer	Insulators	20% mass loss after 21 d	Scaffolds	[46]
PPy	Conducting polymer	Conductors	6–27% mass loss after 80 d	Flexible electronics	[47]
PEDOT:PSS	Conducting polymer	Conductors	100% (in protease solution) mass loss after 4 weeks	Substrate, sensors	[32]

The development and mechanistic understanding of the chemistry of symbiotic bioabsorbable materials determines the application range and future development direction of implantable absorbable electronic systems. In the face of different treatment needs and biomedical situations in practical applications, different time windows are required for symbiotic implant treatment platforms, which also leads to different requirements for symbiotic biomaterials.^[^ ^48^ ^]^ The operational stability and complete bioabsorption periods of implantable devices are the two most important time scales for measuring their functional effectiveness. In general, the time required for the stable operation of the device (usually days or weeks) is much shorter than the time required for complete degradation (usually months or months).^[^ ^49^, ^50^, ^51^ ^]^ Through rational design, the running time of the device can be extended as much as possible to achieve the goal of minimize the difference; however, devices based on bioabsorbable materials inevitably experience physical and structural damage, such as crushing during operation, which leads to differences in practical applications. Figure 3b summarizes the approximate degradation rates of the common bioabsorbable materials. Simultaneously, when using symbiotic implant devices for treatment, avoiding damage and adverse effects on the organism is also a situation. In this section, the classification and progress of materials in the field of bioabsorbable electronic devices are summarized, emphasizing on the degradation mechanisms of inorganic and organic materials and the associated representative chemistry and reaction kinetics.

### 2.1 Symbiotic Bioabsorbable Inorganic Materials

**Figure 4:**
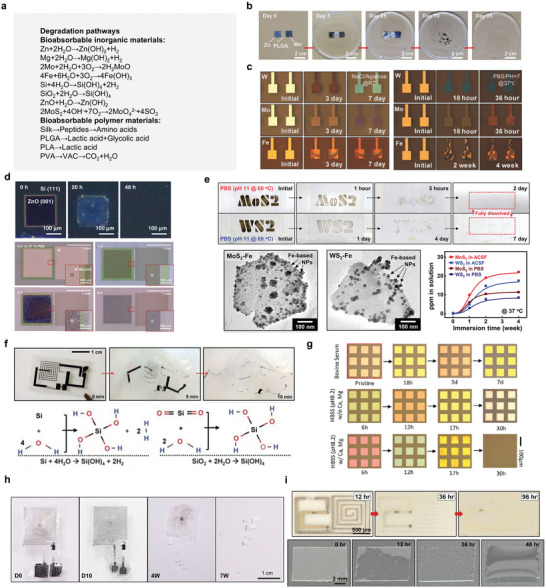
Biodegradation mechanism of inorganic materials. a) Specific degradation reactions of bioabsorbable materials. b) Zn. Reproduced with permission from ref. [52] Copyright 2023, American Chemical Society. c) W, Mo, Fe. Reproduced with permission.^[^ ^24^ ^]^ Copyright 2017, WILEY‐VCH Verlag GmbH & Co. KGaA, Weinheim. d) ZnO. Reproduced with permission.^[^ ^27^ ^]^ Copyright 2019, WILEY‐VCH Verlag GmbH & Co. KGaA, Weinheim. e) MoS_2_, WS_2_. Reproduced with permission.^[^ ^55^ ^]^ Copyright 2022, Wiley‐VCH GmbH. f) Si. Reproduced with permission from ref. [28] Copyright 2012, American Association for the Advancement of Science. g) Si. Reproduced with permission from ref. [58] Copyright 2017, American Chemical Society. h) PLGA in PBS at 37 °C. Reproduced with permission.^[^ ^67^ ^]^ Copyright 2022, American Chemical Society. i) Polyanhydride films with 10% PEG. Reproduced with permission.^[^ ^68^ ^]^ Copyright 2017, The American Association for the Advancement of Science.

Inorganic functional materials, leveraging their mature manufacturing technologies, are extensively used in traditional implanted electronic products. Moreover, these materials are renowned for their stable and superior properties in conducting, semiconducting, and insulating. As research delves deeper into the absorption mechanisms of inorganic symbiotic organisms, their application in bioabsorbable electronics has broadened significantly. Upon exposure to external solution environments or living organisms, these materials undergo hydrolysis, yielding soluble ions. Understanding the interplay between their functional performance and dissolution behavior is crucial. Typically, the dissolution behavior is influenced by both the material's inherent properties and the external environmental conditions. In this section, the types of bioabsorbable inorganic materials and their dissolution mechanisms and methods are introduced. **Figure** **4**a summarizes the degradation mechanisms of common bioabsorbable materials.

#### 2.1.1 Metallic Material

The degradation behavior of metal‐based symbiotic bioabsorbable materials an important parameter index; their degradation follows a series of anode and cathode reactions, usually resulting in a series of complex products. Metals react with the surrounding liquid environment when immersed in water or biological fluids (Figure 3c). For example, electrons are produced through the anodic reaction of metals such as Zn (Figure 4b)^[^ ^52^ ^]^ and Mg^[^ ^53^ ^]^ during water reduction, producing hydrogen and metal hydroxides that are normally deposited on metal surfaces. The surface layer in a biological liquid environment can be eroded by the substances in it (such as chloride or other active ions), resulting in the continuous dissolution of the metal parts. During the dissolution process, owing to their weak reactivity, metals such as Mo, W, and Fe (Figure 4c)^[^ ^24^ ^]^ absorb dissolved oxygen and produce a relative oxygen absorption reaction and a protective layer for the metal. Metals such as Fe form a particularly dense layer when degraded;^[^ ^54^ ^]^ the dense metal layer prevents contact between the solution ions and the metal. The degradation behavior of bioabsorbable metals can be studied from the perspective of electrochemical corrosion. In general, the thermodynamically stable state of a soluble metal is the cation rather than the metal itself. Corrosion is the process by which metals release electrons, produce cations, oxides, and/or hydroxides, and reach their lowest energy levels. It can be seen from the dissolution rate that the more negative the potential value, the more likely it is to lose electrons and dissolve.

At the same time, metal‐derivative materials also occupy a place among absorbable materials. In a biological liquid environment, ZnO can be dissolved in the form of hydroxides or ions and has no effect on organisms. As a simple metal oxide semiconductor, ZnO is widely used in bioabsorbable implantable devices. For example, Figure 4d shows a method for forming a zinc oxide thin film on a Si surface using sputtering technology.^[^ ^27^ ^]^ In some cases, transition metal disulfide compounds exhibit dissolution behavior through hydrolysis. Common examples of such materials are MoS_2_ and WS_2_. The MoS_2_ and WS_2_^[^ ^55^ ^]^ nanosheets prepared by CVD method were dissolved in PBS at the rate of 10–20 nm per day and 5–8 nm per week, respectively, at 60 °C (Figure 4e). As an important symbiotic metal oxide, MgO can be deposited on various substrates via EBPVD and sputtering.^[^ ^56^ ^]^ When exposed to a physiologically relevant fluid, the water molecules trigger a reaction that produces an associated hydroxide. Bioceramic materials, such as calcium orthophosphate, can be effectively degraded in biological fluids owing to their good biocompatibility and pore size.^[^ ^57^ ^]^

#### 2.1.2 Non‐Metallic Inorganic Material

Compared with bioabsorbable metals, bioabsorbable semiconductor materials, which have only recently been extensively studied. Silicon is the most important semiconductor material and is widely used in symbiotic bioabsorbable devices. Silicon is generally considered non‐degradable because it forms a dense layer of silica on its surface in a liquid environment, which makes degradation difficult; however, when the size of silicon is reduced to the nanometer scale, it can be degraded in biological liquids.^[^ ^58^ ^]^ Previous studies have shown that the bioreabsorption of silicon and silicon dioxide involves the formation of orthosilicic acid and hydrogen as products (Figure 4f).^[^ ^28^ ^]^

Liquid environments play an important role in the degradation of symbiotic biomaterials.^[^ ^59^ ^]^ For example, the dissolution rate of silicon nanowires in HBSS and bovine serum solution is significantly higher than that in an ordinary solution environment (Figure 4g) because there are more active ions and bioactive substances in the HBSS and bovine serum solution, which accelerates the degradation process of silicon nanowires.^[^ ^58^ ^]^ By oxygen deposition on the silicon surface, it was found that the overall stability of silicon was greatly improved when there was silicon‐oxygen bonding, which indicates that SiO_2_ has a higher stability to hydrolysis than Si. Cations accelerate the degradation of silicon.

### 2.2 Polymer Material

Polymer materials are crucial for bioabsorbable applications, with a multitude of studies documenting their use in the biomedical field. In this context, we categorize bioabsorbable polymers into two distinct types. The first type comprises materials that can be directly utilized within biological systems, referred to as endogenous materials. The second type encompasses synthetic or modified polymers, which are termed exogenous materials.^[^ ^60^ ^]^

The degradation mechanism of bioabsorbable polymer materials is mainly divided into two parts. The first stage is the process that occurs in vitro, in which polymer macromolecules depolymerize into shorter chains. Generally, there are four main types of reactions: solvated polymer chain interactions with water, enzyme‐catalyzed polymerization, water‐induced depolymerization, and oxidation. During the degradation of polymers, solvated water and long‐chain hydrolysis reactions of molecules are common, whereas enzymatic reactions and induced depolymerization of reactive oxygen species or nitrogen species also occur. Because of the stability of the backbone structure, typical natural polymers can degrade the enzymes adsorbed on the surface of such polymers through enzymatic reactions to produce enzyme‐substrate complexes, which can act as biocatalysts to split the polymer chains and then form small‐molecule reaction products.^[^ ^61^ ^]^ In the second stage, after forming small fragments through mineralization, they are absorbed by the cell (Figure 4d). Depending on the presence of oxygen, biodegradation occurs under two different conditions: aerobic and anaerobic biodegradation. Complete biodegradation or mineralization occurs in the absence of residues; that is, complete conversion of the original product into gaseous and inorganic salt products.^[^ ^62^, ^63^ ^]^

Synthetic polymer materials are widely used in absorbable electronic devices, mainly because the properties of endogenous polymer materials such as high electrical conductivity and high ion storage capacity can be achieved through artificial design methods. The insulation of synthetic materials is extremely important.^[^ ^64^, ^65^ ^]^ PVA can be used as a bioabsorbable substrate.^[^ ^66^ ^]^ When PVA enters the body, it undergoes a series of reactions, including the expansion and repeated oxidation of microorganisms, enzymatic hydrolysis of hydrolytic enzymes, and the formation of simple byproducts. Enzymatic hydrolysis and oxidation are dominant during this degradation process. Polymers are mainly hydrolyzed to form small molecules, which are then taken up by the cells. For example, the degradation rate of PLGA could be altered by changing the ratio of lactic acid to glycolic acid (Figure 4h). Use in PBS, pH 7.4, 37 °C; Ratios of 75:25, 65:35, and 50:50 showed complete degradation within 50, 40, and 30 d.^[^ ^67^ ^]^ Another example is a PA‐based polymer film that has stable mechanical properties in flexible systems and acts as a substrate to quickly dissolve anhydrides and hydrolyze carboxylic acids in ambient air with sufficient humidity (Figure 4i).^[^ ^68^ ^]^ By adding 1,4‐butanedithiol, the dissolution rate of PA‐based materials (PBS; pH 7.4, 37 °C) 10–15 times slower. The thickness decreased linearly (1.3 µm per day), indicating hydrolytic surface erosion of PA.^[^ ^69^ ^]^

## 3 Bioabsorbable Electronic Components

A wide range of bioabsorbable electronic components can be fabricated using symbiotic bioabsorbable materials. This section focuses on different types of implantable symbiotic bioabsorbable devices, as well as their operating principles and performance.

### 3.1 Bioabsorbable Sensor

#### 3.1.1 Physical Sensor

**Figure 5:**
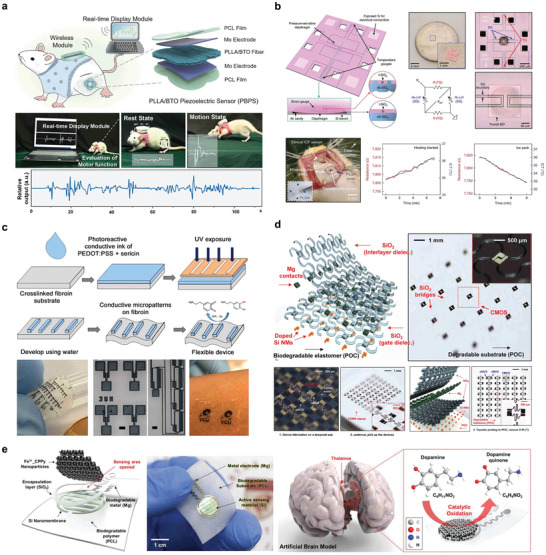
Bioabsorbable sensors. a,b) Bioabsorbable physical sensor; c–e) Bioabsorbable chemical sensor. a) Schematic of PBPS in rats and photos of wireless assessment of motor function. Reproduced with permission.^[^ ^70^ ^]^ Copyright 2024, Wiley‐VCH GmbH. b) Schematic diagram of a bioabsorbable pressure sensor composed of monocrystalline silicon and silica layers and images of intracranial temperature and pressure monitoring in rats. Reproduced with permission.^[^ ^71^ ^]^ Copyright 2018, Springer Nature. c) Large‐area micropattern PEDOT: PSS process flow and specific device pictures. Reproduced with permission from ref. [32] Copyright 2016, Elsevier B.V. d) Schematic decomposition of transient silicon CMOS devices. Reproduced with permission.^[^ ^72^ ^]^ Copyright 2015, American Chemical Society. e) Biodegradable neurotransmitter detection system and in vivo test diagram. Reproduced with permission.^[^ ^73^ ^]^ Copyright 2018, WILEY‐VCH Verlag GmbH & Co. KGaA, Weinheim.

In principle, physical sensors are classified into powerful electric, thermoelectric, photoelectric, and capacitive sensors. The most common type of symbiotic bioabsorbable sensor is a pressure sensor, which is currently the most widely used implantable absorbable sensor. This sensor transmits signals by measuring the pressure changes in tissues or organs to monitor physiological signals. The phase transition of the diaphragm caused the piezoresistive sensor to produce a signal change. Polylactic acid (PLLA) can exhibit certain piezoelectric properties through a specific preparation method, and doping BTO NPs in PLLA increases the likelihood of generating potentials in more directions throughout the material, thereby improving the piezoelectric output.^[^ ^70^ ^]^ Thus, a symbiotic bioabsorbable implantable PLLA/BTO piezoelectric sensor (PBPS) was developed by doping PLLA fibers with BTO, which could be used to monitor motor function recovery after nerve injury. PBPS can be implanted using ordinary tissue scaffolds. The PBPS can be implanted using common tissue stents (**Figure** **5**a).

Bioabsorbable triboelectric sensors can directly convert changes in the ambient pressure into electrical signals. One study reported a bioabsorbable pressure sensor that utilizes four silicon NM‐based sensors as a strain and thermometer, with heat‐grown silica layers as an envelope (Figure 5b), forming a fully degradable platform that extends the functional life of Si devices by using t‐SiO_2_ layers to protect them from biological fluids and can be used to monitor intracranial pressure in rats. The device monitors specific physiological signals by extending the strain gauge into the gas chamber to form a floating pressure‐sensitive diaphragm. As the pressures of the surrounding environment and air in the chamber change, the piezoresistive response of the strain gauge produces an output.^[^ ^71^ ^]^

#### 3.1.2 Chemical Sensor

Physiological signals generated by chemical reactions in biological systems are also important aspects of symbiotic biosensors. Symbiotic bioabsorbable pH sensors have been proposed because chemicals in living organisms are affected by pH. A pH‐sensitive sensor was developed based on an amine/oxide‐functionalized Si NR field‐effect transistor.^[^ ^72^ ^]^ (Figure 5d). The different functionalizations of the functional groups in different PH liquid environments can affect the charge state of the device to monitor changes in the physiological environment.

Dopamine transmitters play an important role in the regulation of brain physiological activity, which controls the brain's behavioral reward mechanism. Neurotransmitter levels were detected using dopamine‐sensitive chemical sensors as active sensing elements (Figure 5e). Dopamine molecules chemically bonded with Fe^3+^_CPPy NPs to form dopamine–quinone compounds, which in turn produced electrons that were transferred to Si nanometers, producing a measurable electric current.^[^ ^73^ ^]^ The dopamine concentration can also be monitored by electrochemical methods, such as fixing glucose oxidase by bonding to the PEDOT:PSS working electrode (Figure 5c).^[^ ^32^ ^]^ The detection of protein coupling reactions is also an important aspect of bioabsorbable chemical sensors. One example is the preparation of a black phosphorus‐based field‐effect transistor (FET) biosensor using a synthetic gold antibody conjugate label on a black phosphorus nanosheet. It uses a mechanically stripped BP nanosheet as the sensing/conducting channel of the FET, an Al_2_O_3_ film as the surface‐passivated dielectric layer, and an antibody probe bound to gold nanoparticles. The sensor response was measured based on the change in resistance caused by the introduction of the antigen to the BP. The results showed that BP exhibited excellent performance as a sensing channel for FET biosensor applications.^[^ ^74^ ^]^

### 3.2 Symbiotic Bioabsorbable Energy Storage Device

Because some implantable devices still need to provide energy through external energy supply devices in the body, energy storage devices prepared using the symbiotic bioabsorbable materials described in Section 2 are also important. **Table** **2** summarizes the types and energy densities of recent symbiotic energy‐storage devices.

**Table 2:** Symbiotic absorbable energy storage device.

Electrode materials	Electrolyte	Voltage windows (V)	Specific capacitance	Power density (mW cm^−2^)	Energy density	Refs.
MoO_x_ flakes	Sodium alginate gel	1.0	112.5 mF cm^−2^	2.53	15.64 µWh cm^−2^	[75]
MoS_2_/Zn	Alg‐Zn gel	1.1	93.5 mF cm^−2^	1.97	1.65 µWh cm^−2^	[76]
Fe/ZnO	PVA/PBS hydrogel	1.0	1.1 mF cm^−2^	0.526	0.153 µWh cm^−2^	[77]
Mo wire	PVA /NaCl gel	0.8	4.15 mF cm^−2^	0.8	0.37 µWh cm^−2^	[78]
SrMA/A‐rGo hydrogel	PVA gel	1.0	0.1 mF cm^−2^	0.026	0.014 µWh cm^−2^	[79]
Zn@ppy	NaCl/ agarose gel	0.9	2.84 mF cm^−2^	0.38	0.394 µWh cm^−2^	[80]
MnO_2_@Si NWs	Li‐doped ionic liquid	2.2	13 mF cm^−2^	0.388	9.1 µWh cm^−2^	[81]
Metallic Mo	Agarose gel	0.8	1.6 mF cm^−2^	1	0.14 µWh cm^−2^	[82]
ZnO nanowire	PVA/H_3_PO_4_ gel	0.8	2.4 mF cm^−2^	0.014	0.027 µWh cm^−2^	[83]
Ferritin MWNT fiber	PBS/NaCl	0.8	32.9 mF cm^−2^	0.150	0.82 µWh cm^−2^	[84]
Mo/C/SA	PVA/IL	1.5	65 mF cm^−2^	1.1	19 µWh cm^−2^	[85]
MoO_3_–MoS_2_/Zn	Gelatin/ZnSO_4_ gel	1.1	181.86 mF cm^−2^	2.2	30.56 µWh cm^−2^	[86]
Mg−Mo	PBS	0.45	276 mAh g^−1^	N/A	2.2 Ah g^−1^	[87]
Mg−Fe	0.9 wt.% NaCl	0.7	1100 mAh g^−1^	N/A	694 Wh Kg^−1^ kg^−1^	[88]
Mg−I_2_	IL/aqueous	1.8	9.8 mAh cm^−2^	0.7 mW cm^−2^	17.7 mWh cm^−2^	[89]
Mg‐MoO3	calcium alginate gel	1.5	6.5 mAh cm^−2^	N/A	1.72 mWh cm^−2^	[90]
Zn‐(PEDOT‐COOH)	gelatin−ZnSO4 gel	1.2	31.8 mAh g^−1^	N/A	N/A	[91]

#### 3.2.1 Battery

Due to their high energy density and output voltage, batteries have emerged as the premier energy storage solution in contemporary society. Consequently, they are often the preferred choice for researchers when considering energy storage in implantable devices.^[^ ^92^, ^93^, ^94^, ^95^, ^96^, ^97^ ^]^ A battery typically consists of four main components: the anode, cathode, electrolyte, and separator. For use in implantable devices, an additional packaging layer is usually incorporated. This layer is designed to harness chemical energy through a spontaneous redox reaction, thereby converting it into electrical energy to power the treatment of biological tissues.^[^ ^52^, ^98^ ^]^

**Figure 6:**
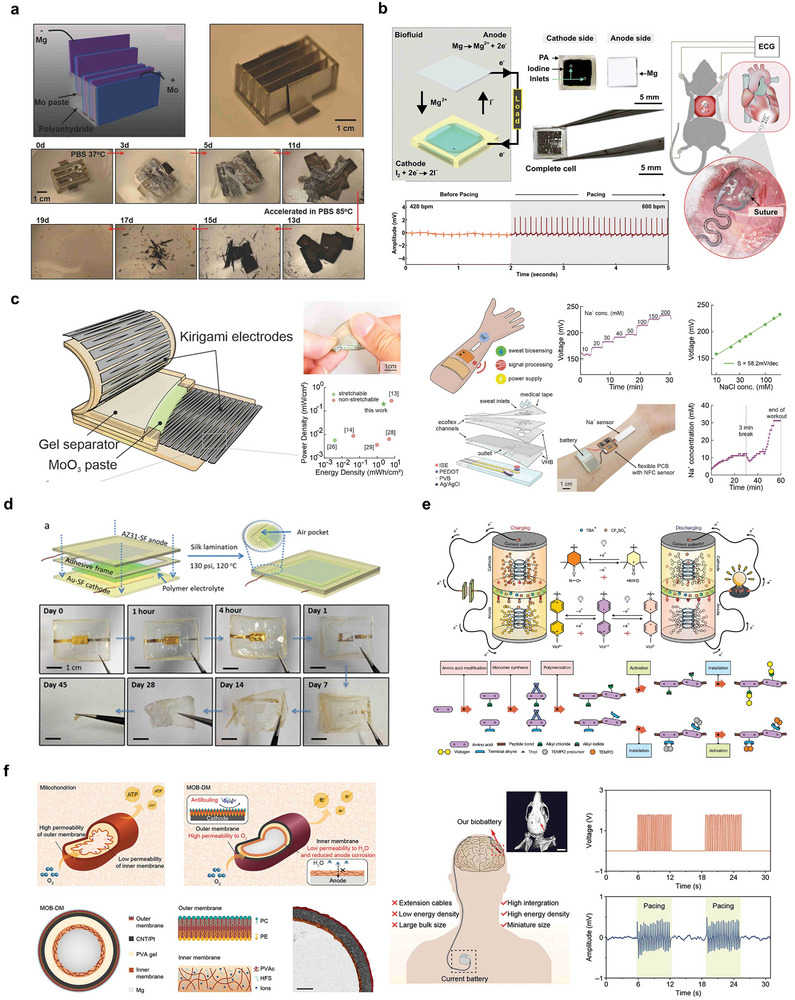
Bioabsorbable batteries. a) Structure and optical image of four series Mg‐Mo cells and dissolution behavior. Reproduced with permission.^[^ ^87^ ^]^ Copyright 2014, WILEY‐VCH Verlag GmbH & Co. KGaA, Weinheim. b) Structure diagram and in vivo experiment of Mg‐I_2_ battery with two electrolytes. Reproduced with permission.^[^ ^89^ ^]^ Copyright 2022, The Royal Society of Chemistry. c) Structure diagram and degradation behavior of degradable MoO_3_‐Mo battery. Reproduced with permission.^[^ ^100^ ^]^ Copyright 2022, Wiley‐VCH GmbH. d) Structure diagram and degradation behavior of polymer electrolyte battery. Reproduced with permission.^[^ ^101^ ^]^ Copyright 2017, American Chemical Society. e) Polypeptide organic radical cell and synthesis method. Reproduced with permission.^[^ ^98^ ^]^ Copyright 2021, Springer Nature. f) Structure diagram and in vivo demonstration of MO‐DM cell with imitation mitochondria structure. Reproduced with permission.^[^ ^103^ ^]^ Copyright 2023, Wiley‐VCH GmbH.

The first reported bioabsorbable battery design used Mg foil as the anode and Fe, W, or Mo foil as the cathode.^[^ ^87^ ^]^ The selected battery configuration has an output voltage of 0.45 V, which could be powered for up to 4 h in vivo and had an energy density of 276 mAh g^−1^ (**Figure** **6**a).

In this case, the energy output of the Mg–Mo battery is not high, and further modification of the battery structure can make the performance of a highly implantable battery more in line with the demand, among which the Mg–I_2_ degradable system made of an IL/water dual‐electrolyte structure system can effectively improve the battery performance (Figure 6b). In a small animal model, the battery was able to work continuously in vivo for 5 d, after which the capacity decreased from 15 to 0.6 mAh cm^−2^ owing to the degradation of magnesium itself in vivo.^[^ ^89^ ^]^ These batteries also exhibit potential as pacemakers, sensors, and heaters for heat therapy and wireless environmental monitoring. Cathode materials such as MoO_3_ provide a higher electrochemical potential to mitigate the voltage output and energy density.^[^ ^99^ ^]^ The cathode reaction is MoO_3_ + αMn^+^ + nαe^−^→MnMoO_3_. A typical bioabsorbable MG‐MoO_3_ battery^[^ ^100^ ^]^ can achieve an output voltage of up to 1.6 V (Figure 6c). By combining inherent stretchability with engineered stretchability, high‐power biodegradable batteries for sustainable and stretchable electronics can be manufactured by creating reversible stretchable metal electrode kirigami patterns. The use of these electrodes in biodegradable batteries can produce batteries with an energy density of up to 1.72 mWh cm^−2^ that can withstand deformations of up to 35% in a single direction, such as bending, twisting, and stretching, without significant performance loss. The use of endogenous macromolecules as packaging and electrolytes is also a major direction for absorbable batteries. One example of this is the use of biodegradable polymer electrolytes and silk packaging layers. The resulting PE composite had an ionic conductivity of 3.4 mS cm^−1^ and exhibited 2 d degradation in a concentrated buffered protease solution (Figure 6d).^[^ ^101^ ^]^ By plating a layer of Mo_2_C on the Mo electrode, the output voltage of the Mg Mo_2_C battery can be increased to 1.4 V, twice that of the Mo cathode, and the battery can work as a wireless sensor module.^[^ ^102^ ^]^

Endogenous macromolecules can also be used as sources of chemical energy for batteries. Anode and cathode materials composed of the redox‐active amino acid macromolecules viologen and biTEMPO, respectively, were synthesized by ring‐opening polymerization.^[^ ^98^ ^]^ Practical tests of the polypeptide battery demonstrated a capacity of 37.8 mAh g^−1^, while the battery composition is fully degradable into human nutrients (Figure 6e). Another type of battery builds a double‐membrane structure by imitating the structure of cell mitochondria, which uses a highly hydrophobic polymer layer to prevent the penetration of liquids and reduce the corrosion rate of the anode material, whereas the outer membrane uses a modified phospholipid layer to ensure the continuous migration of oxygen (Figure 6f).^[^ ^103^ ^]^

#### 3.2.2 Supercapacitors

Supercapacitors are electrochemical capacitors that have a lower output voltage than batteries but extremely high energy density and cycle life, making them another choice in the field of energy storage.^[^ ^104^ ^]^ A symbiotic bioabsorbable supercapacitor consists of three parts: electrode material, electrolyte, and separator. Supercapacitors can be divided into three main types: electric double‐layer, pseudocapacitor, and hybrid supercapacitors.^[^ ^105^ ^]^

**Figure 7:**
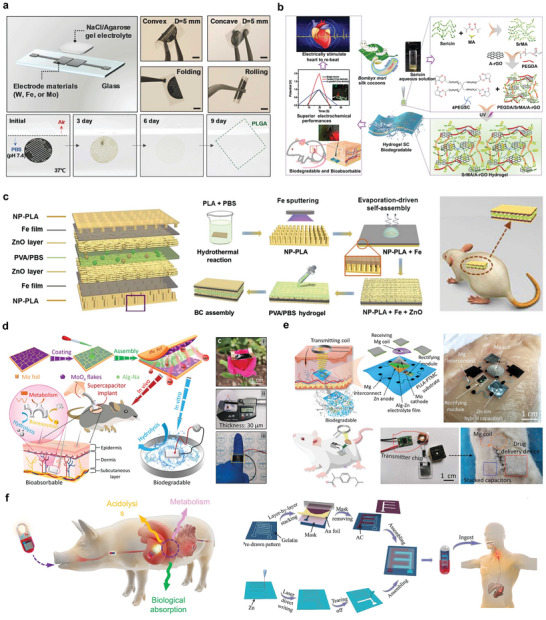
Bioabsorbable supercapacitors. a) Schematic diagram and degradation behavior of a capacitor composed of a biodegradable metal film electrode and NaCl/Agarose gel electrolyte. Reproduced with permission.^[^ ^24^ ^]^ Copyright 2017, WILEY‐VCH Verlag GmbH & Co. KGaA, Weinheim. b) Schematic diagram of the manufacturing process and application of supercapacitors based on SrMA/A‐rGO hydrogel electrodes. Reproduced with permission.^[^ ^106^ ^]^ Copyright 2023, Wiley‐VCH GmbH. c) Structure, preparation process diagram, and implantation demonstration of biodegradable supercapacitor. Reproduced with permission.^[^ ^107^ ^]^ Copyright 2019, WILEY‐VCH Verlag GmbH & Co. KGaA, Weinheim. d) Diagram of Mo‐MoO_x_ electrode sheet and Alg‐Na gel electrolyte supercapacitor. Reproduced with permission.^[^ ^110^ ^]^ Copyright 2021, The American Association for the Advancement of Science. e) Schematic diagram and in vivo experimental demonstration of Zn‐MoS_2_ supercapacitor. Reproduced with permission.^[^ ^111^ ^]^ Copyright 2023, The American Association for the Advancement of Science. f) Schematic diagram of the manufacturing process of an edible Zn‐ion‐based MSC. Reproduced with permission.^[^ ^115^ ^]^ Copyright 2022, American Chemical Society.

The first reported bioabsorbable supercapacitor was a double electric layer capacitor containing W, Fe, or Mo planar electrodes on the PLGA substrate of an agar‐gel electrolyte (**Figure** **7**a). Cyclic voltammetry (CV) measurements showed that the performance remained the same under different deformations and exhibited better reversible capacitance behavior than similar devices constructed with Au.^[^ ^24^ ^]^

Another example of a biodegradable supercapacitor is the formation of a multinetwork conductive electrode by crosslinking amine‐reduction‐oxide‐graphene methacrylate‐modified sericin (SrMA/A‐RGO) (Figure 7b).^[^ ^106^ ^]^ Bacterial cellulose (BC) plays an important role as a fully degradable biological material in SCs. Nanocolumns were prepared on the surface of a polylactic acid support substrate as an adhesion promoter for the Fe film. Polylactic acid (NP‐PLA) with nanocolumns provides a stable substrate for device attachment. The supercapacitor uses a nanoporous zinc oxide layer as the electrode material for ion storage and a PBS/PVA hydrogel as the electrolyte and separator.^[^ ^107^ ^]^ In addition, BC achieved a high voltage of 1.5 V for 30 d and a long working time of 50 d in PBS and SD rats, respectively. In liquid environments, BCS can operate anywhere from a few days to a few weeks by adopting different edge encapsulation strategies (Figure 7c). Protein‐based supercapacitors are highly biocompatible. Rusling et al. reported a symbiotic supercapacitor prepared using bGO and myoglobin.^[^ ^108^ ^]^ Using human biofluids as electrolytes, a capacitance of up to 534 F cm^−3^ at 2.5 A g^−1^ was achieved. In addition, the team reported a graphene‐myoglobin symbiotic bioabsorbable supercapacitor prepared based on solution reduction methods,^[^ ^109^ ^]^ which can be used to store the collected energy and power of the DBS.

However, the disadvantage of these examples is that their energy and/or power densities are relatively low. High‐performance pseudocapacitor supercapacitor implants can be obtained by synthesizing 2‐D, defective amorphous molybdenum oxide (MoOx) sheets directly on water‐soluble molybdenum foils as a binder using an all‐green, controllable electrochemical oxidation method, which can biodegrade and bioabsorb in animals (Figure 7d).^[^ ^110^ ^]^ This fully biodegradable supercapacitor implant had a high surface capacitance and excellent energy density.^[^ ^123^ ^]^Another implantable degradable capacitor based on the principle of pseudocapacitance uses molybdenum sulfide nanoparticles as the cathode, an ion‐crosslinked alginate gel as the electrolyte, and Zn foil as the anode, which has a high output voltage.^[^ ^111^ ^]^ Hybrid capacitors can be used simultaneously without charge. A Voc of 1.022 V was generated at the two electrodes, and a small bulb could be easily lit (Figure 7e). The structure integrates a wireless charging module with an energy storage module (series zinc ion supercapacitor), which can output DC voltage instantaneously and provide continuous power for a long time, thereby maximizing the advantages of pseudocapacitor supercapacitors. Using MXene 2D material, binderless flexible films can be prepared as flexible solid‐state biodegradable supercapacitors (FSBSCS), which have high energy density and good biocompatibility.^[^ ^112^ ^]^

At the same time, advancements have been made to prepare edible biodegradable supercapacitors by using food supplements, additives, or explicit foods.^[^ ^113^, ^114^, ^115^ ^]^ Figure 7f shows an edible supercapacitor that combines edible activated carbon, a Zn microanode, and a ZnSO_4_ electrolyte.^[^ ^115^ ^]^ Zn‐based MSC was prepared using e‐AC as the microcathode, Zn foil as the microanode, and ZnSO_4_ gel as the electrolyte.

### 3.3 Symbiotic Bioabsorbable Energy Harvesting Device

Symbiotic bioabsorbable energy‐harvesting systems based on piezoelectric or triboelectric effects can harvest microenergy within the body and use it to power electronic devices. **Table** **3** outlines the performance characteristics of symbiotic bioabsorbable energy‐harvesting systems.

**Table 3:** Performance characteristics of symbiotic bioabsorbable energy harvesting systems.

Materials	Type	Energy sources	Voltage and current outputs	Generated power	Refs.
ZnO	Piezoelectric	Cyclic bending	≈1.14 V and ≈0.55 nA	≈10 nW cm^−2^	[116]
KNN/PLLA/PHBV	Piezoelectric	Ultrasound (0.7W cm^−2^)	12 V and 36 µA	0.3 mW cm^−2^	[117]
KNN/PLA	Piezoelectric	Acoustic pressure (150 kPa)	12.09 V and 20.8 µA	N/A	[38]
PLA/gelatin	Triboelectric	contact force (50 N)	500 V and 10.6 mA m^−2^	5 W m^−2^	[118]
PLGA/PCL	Triboelectric	compression/release (1 Hz)	≈40 V and ≈1 µA	32.6 mW m^−2^	[119]
PLGA/Mg	Triboelectric	Rat's normal movements	≈2 V	N/A	[119]
Mg/PHBV/PEG	Triboelectric	ultrasound (0.5W cm^−2^)	4.51 V and 27.86 µA (20 kHz)	17.24µW cm^−2^	[120]
Mg/SF	Radio Frequency	≈20 W	5% (15 mm distance)	54 mW	[121]
Mg/PLGA	Radio Frequency	≈12 W	15.7%	100 mW	[15]

#### 3.3.1 Symbiotic Bioabsorbable Friction Nanogenerator

Bioabsorbable energy‐harvesting devices are primarily friction nanogenerators (DB‐TENG) based on bioabsorbable materials, which have shown unique advantages in the collection of bioenergy.^[^ ^122^ ^]^ The attempt to apply degradable materials in TENG began in 2015, when Valentini first applied plant‐based material alginate and graphene oxide composite membranes to TENG.^[^ ^123^ ^]^ In 2016, the concept of degrading TENG was first proposed by Zheng Qiang et al., who reported the use of artificial biodegradable materials as triboelectric layers to manufacture TENG, proposed the concept of “biodegradable and implantable TENG” for the first time, and implanted the fully biodegradable DB‐TENG into the human body for medical care.^[^ ^124^ ^]^

**Figure 8:**
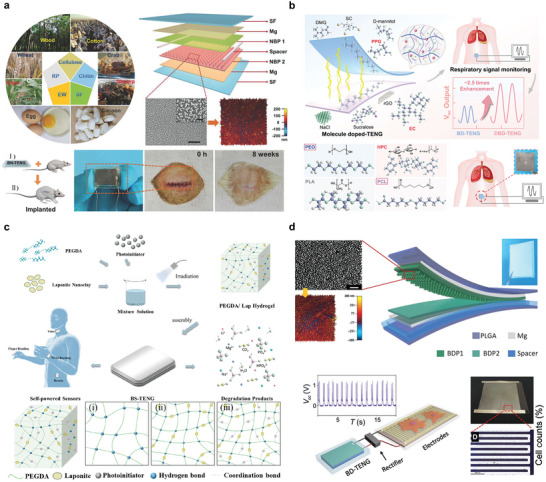
Bioabsorbable friction nanogenerator. a) Common bioabsorbable material sources, BN‐TENG device structure diagram, and in vivo experiment diagram. Reproduced with permission.^[^ ^125^ ^]^ Copyright 2018, WILEY‐VCH Verlag GmbH & Co. KGaA, Weinheim. b) Schematic diagram, synthesis method of DBD‐TENG, and its application in respiratory signal monitoring. Reproduced with permission.^[^ ^126^ ^]^ Copyright 2024, Wiley‐VCH GmbH. c) Preparation process, design principle, and application in monitoring physiological signals of BS‐TENGs of PEGDA/Lap hydrogel. Reproduced with permission.^[^ ^129^ ^]^ Copyright 2023, American Chemical Society. d) Schematic diagram of BD‐TENG prepared by PLGA, PHB/V, PCL, and PVA and demonstration of nerve stimulation. Reproduced with permission.^[^ ^124^ ^]^ Copyright 2016, The American Association for the Advancement of Science.

Various fully bioabsorbable natural materials can be used as the basis for triboelectric nanogenerators (BN‐TENGs), and various triboelectric outputs of these natural materials can be achieved through the combination of a single material and their pairs. The tribological series of these materials was comprehensively studied for the first time,^[^ ^125^ ^]^ which greatly promoted the development of natural materials applied to friction electrical parts such as TENG (**Figure** **8**a). The BN‐TENG can be completely degraded and absorbed in SD rats, avoiding side effects such as secondary surgery. For ordinary natural materials, the output performance is still low, and molecular doping is a simple and effective way to change the triboelectric polarity of triboelectric materials and improve the output performance of bd‐tengs. By selecting different types of doping molecules for positive and negative friction materials, it is important to study the triboelectric polarity and its related rules (Figure 8b). By doping with PPG and EC, the output performance of the BD‐TENG was improved, and the device exhibited good sensitivity for respiratory signal monitoring.^[^ ^126^ ^]^ Using inactivated bacterial membranes as triboelectric materials, a simple, fast, and efficient fabricated bacterial membrane TENG (BF‐TENG) can be prepared,^[^ ^127^ ^]^ which has a stable electrical output, good durability and fatigue resistance, and has excellent superior capabilities along the contact and non‐contact sensing. By combining biomacromolecules with triboelectric materials, the output performance of TENG can be effectively improved. By introducing β‐lactoglobulin fibril (BF) into the PVA aerogel network, the mechanical properties of BF‐PVA porous films are significantly enhanced, and the voltage output of BF‐PVA in triboelectric performance tests is also increased to 203 V.^[^ ^128^ ^]^

A PEGDA/Lap nanocomposite hydrogel was prepared using PEGDA and laterite, and a completely degraded single‐electrode triboelectric nanogenerator was constructed.^[^ ^129^ ^]^ The composite hydrogel has an extremely high strain capacity, and the BS‐TENG produces an open‐circuit voltage of 10.4 V in single‐electrode mode, which can stably operate 106 times (Figure 8c). The device can be degraded in a controlled manner without causing harm. By assembling two selected BDP layers with nanoscale surface structures as friction parts, a fully bioabsorbable friction nanogenerator (BD‐TENG) was constructed by placing a BDP spacer layer between these friction layers, depositing a magnesium thin film on one side of the friction layer as an electrode layer, and packaging with BDP (Figure 8d).^[^ ^124^ ^]^

#### 3.3.2 Symbiotic Bioabsorbable Piezoelectric Materials

**Figure 9:**
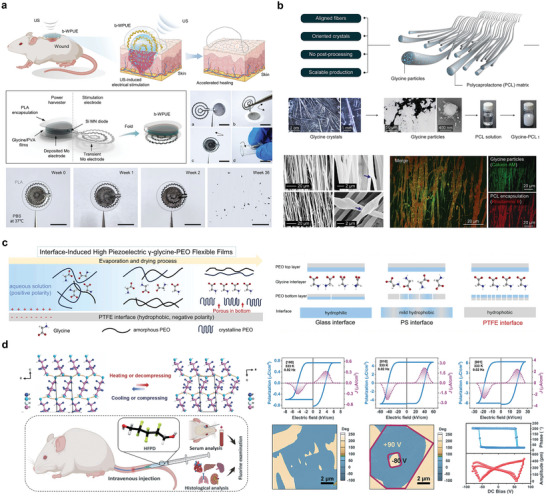
Bioabsorbable piezoelectric materials. a) Structure diagram and in vivo experimental demonstration of b‐WPUE. Reproduced with permission.^[^ ^131^ ^]^ Copyright 2024, The American Association for the Advancement of Science. b) Structure diagram and microscopic characterization of flexible amino acid nanofibers. Reproduced with permission.^[^ ^132^ ^]^ Copyright 2023, The American Association for the Advancement of Science. c) Synthesis method of γ‐glycine‐PEO piezoelectric materials and schematic diagram of different interface‐induced orientations. Reproduced with permission.^[^ ^133^ ^]^ Copyright 2023, Elsevier Ltd. d) Schematic diagram of ferroelectric molecule crystal structure, specific properties and in vivo experimental demonstration. Reproduced with permission.^[^ ^134^ ^]^ Copyright 2024, The American Association for the Advancement of Science.

Piezoelectric materials refer to materials can spontaneously generate electrical signals under pressure. Because the pressure in organisms is complex and changeable, symbiotic bioabsorbable piezoelectric materials are an important choice for electrical stimulation therapy devices.^[^ ^130^ ^]^ **Figure** **9**a shows a biodegradable injectable piezoelectric hydrogel made from frozen sliced piezoelectric short nanofiber PLLA and collagen matrix. The peak output voltage of the NFsPLLA hydrogel scaffolds in the dry state was ≈33.7 mV, which induced cartilage formation in the in vitro experiments. In vivo experiments in rabbit models showed that the modified hydrogel could treat severe OC defects, demonstrating its excellent bone repair ability.^[^ ^131^ ^]^

Biodegradable piezoelectric materials can also be prepared by combining amino acids with piezoelectric properties, using piezoelectric polymers. Figure 9b describes a method for embedding highly piezoelectric glycine crystals into a PCL polymer matrix to construct a stable piezoelectric performance of glycine‐polycaprolactone film. The piezoelectric coefficient d_33_ of the glycine‐PCL composite has a high performance of 19 pC N^−1^. Controlled in vivo release of blood‐brain barrier‐blocking drugs can be achieved using animal tail vein models. Additionally, piezoelectric fiber materials can be utilized for the release of therapeutic drugs targeting tumors in nude mouse models.^[^ ^132^ ^]^ Another example is the synthesis of γ‐glycine with PEO to prepare a high‐voltage flexible biodegradable film (Figure 9c). The film had a high out‐of‐plane piezoelectric coefficient (d_33_) of ≈8.2 pC N^−1^ at the highly hydrophobic PTFE interface.^[^ ^133^ ^]^

Additionally, some biodegradable molecular crystals exhibit strong piezoelectric responses. Figure 9d shows the biodegradable ferroelectric molecular crystal HOCH_2_ (CF_2_) _3_CH_2_OH with a high piezoelectric response. Here, d_33_ reaches ≈138 pC N^−1^ and the piezoelectric voltage constant g_33_ is ≈2450 × 10 ^−3^ Vm N^−1^ under non‐polarized conditions. The material had no side effects in SD rats.^[^ ^134^ ^]^

Radio frequency electromagnetic transmission serves as a wireless power‐delivery method, capable of directing energy input to deep‐tissue locations through near‐ or far‐field mechanisms. The integration of bio‐wireless transmission eliminates the need for a second surgery to replace energy modules, thereby reducing the burden on patients. Reports detail the design and manufacture of a magnesium‐based wireless microheater utilizing ion beam etching (IBE) technology; this frequency‐selective microstructure functions as both an energy receiver and a microheater for biodegradable implantable medical devices.^[^ ^135^ ^]^ Another magnesium‐based RF wireless transmission system consisted of two Mg coils in series with a PLGA layer that exhibited photoelectric stimulation properties.^[^ ^136^ ^]^

### 3.4 Symbiotic Bioabsorbable Devices Circuit

The symbiotic bioabsorbable materials described in Section 2 can form circuit components with different functions through various combinations. This section focuses on the different classes of such components, mechanisms, and performance; examples include different symbiotic bioabsorbable circuit components, such as transistors and diodes.

#### 3.4.1 Symbiotic Bioabsorbable Transistors

Transistors are one of the most critical components of modern electrical appliances that can control the on‐off state of other components by judging the size of the current and choosing whether the current flows. In symbiotic bioabsorbable devices, transistors are an important component that provides the ability to achieve controllable treatment of devices for implantable devices.

**Figure 10:**
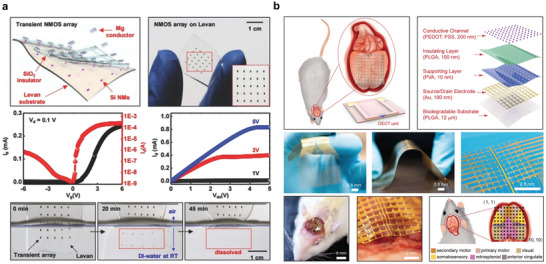
Symbiotic bioabsorbable transistors. a) Structure diagram and degradation behavior of transient silicon‐based N‐channel transistor array. Reproduced with permission.^[^ ^137^ ^]^ Copyright 2018, WILEY‐VCH Verlag GmbH & Co. KGaA, Weinheim. b) Schematic diagram of OECT array structure and demonstration as brain‐computer interface. Reproduced with permission.^[^ ^138^ ^]^ Copyright 2023, Wiley‐VCH GmbH.

The first transistor, which is made of a symbiotic biomaterial, integrates silicon on a fibrin substrate to control the current input. Thin flexible Si nanotubes were constructed on a polyimide substrate and subsequently transferred to a separate silk film to produce a partially bioabsorbable transistor system.^[^ ^14^ ^]^ Synthetic symbiotic bioabsorbable polymers can also be used as substrates for transistors and devices, such as synthesizing Si nanotransistor arrays as transistors on PLGA, PLA, and PCL.^[^ ^137^ ^]^ These devices exhibit excellent performance, with a gain of ≈80 and threshold voltage of ≈−1 V (**Figure** **10**a). Organic and polymeric materials also serve as bases for various types of bioabsorbable electronic devices. For example, by constructing a PLGA insulation layer and an Au electrode on a PEDOT:PSS flexible substrate membrane, an ultrathin, lightweight, and flexible multichannel neural interface based on an organic electrochemical transistor (OECT) network was fabricated and encapsulated by a PVA membrane, which has continuous high‐fidelity neural signal mapping and the ability to actively degrade biosafety after performing the function. Simultaneously, the platform provided a uniformly high transconductance of up to 9.0 mS in vitro and recorded enhanced EP signals with a signal‐to‐noise ratio of up to 37 dB in vivo (Figure 10b). Implantable OECT arrays are excellent tools for establishing stable functional neural interfaces designed as fully biodegradable in vivo electronic platforms.^[^ ^138^ ^]^

#### 3.4.2 Symbiotic Bioabsorbable Diode

As important devices in the traditional electronics field, diodes are used to limit the flow of current in only one direction. The P–N diode is a connection between P‐ and N‐type semiconductors; the potential at the interface of the P‐ and N‐type semiconductors is different only when a potential difference beyond the interface is applied.

**Figure 11:**
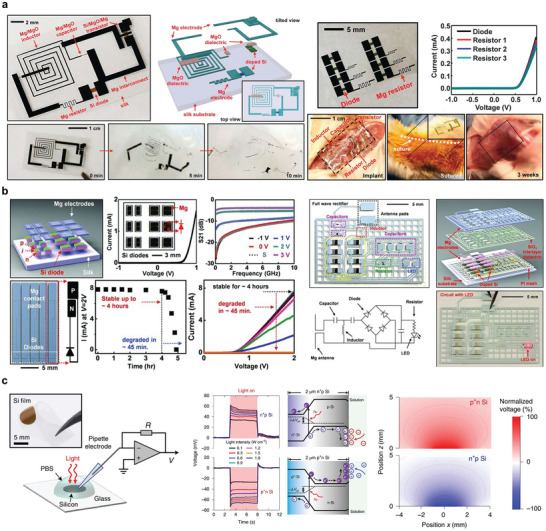
Symbiotic bioabsorbable diode. a) Schematic diagram of device structure and degradation behavior. Reproduced with permission.^[^ ^28^ ^]^ Copyright 2012, The American Association for the Advancement of Science. b) Structure diagram and dissolution kinetics of transient diode. Reproduced with permission.^[^ ^121^ ^]^ Copyright 2013, WILEY‐VCH Verlag GmbH & Co. KGaA, Weinheim. c) Photoelectric effect and principle of thin‐film silicon diode. Reproduced with permission.^[^ ^139^ ^]^ Copyright 2022, Springer Nature.

Silicon nanodiodes can be fabricated by introducing p‐ and n‐type dopants into the lattice. An example is the use of p–n junctions with Mg electrodes on silk substrates (**Figure** **11**a).^[^ ^28^ ^]^ The doping structure involves an inherent region between n‐ and p‐doped regions, resulting in a PIN diode. The switching voltage is ≈0.7 V, and at 1 V, the rectification responds to the corresponding high‐frequency region (Figure 11b) when manufactured using an improved version of the technology developed for the semiconductor industry.^[^ ^121^ ^]^

Another example is the injection of boron and phosphorus ions into N‐ and P‐type insulator silicon wafers, where different junctions (P+N si and N+P si) can be formed, and the film pattern can be defined using lithography, selective etching, and transfer printing to make independent Si films (Figure 11c). Heterogeneous integration into rigid substrates. Planar P+N and N+P si films are suitable for regulating large areas of nerve cells or tissues at the millimeter scale.^[^ ^139^ ^]^

## 4 Symbiotic Bioabsorbable Integrated Systems and Biomedical Applications

**Figure 12:**
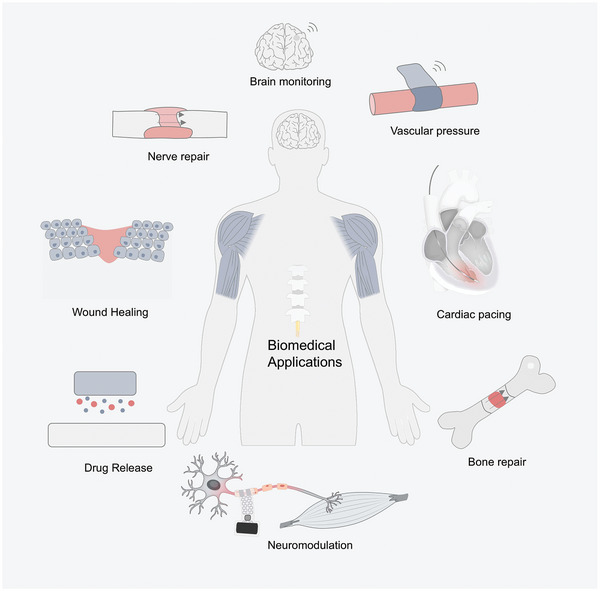
Application of symbiotic bioabsorbable devices.

Symbiotic bioabsorbable system integration applications form a therapeutic platform by integrating the different device combinations described in Section 3. The biomedical applications of these bioabsorbable electronics encompass a range of biotherapy systems, including diagnostics, tissue repair, and cardiac pacing, along with integrated systems for brain and blood monitoring, as well as opportunities in biomedical advancements, tissue regeneration, cardiac stimulation, and pharmaceutical therapy. **Figure** **12** depicts the pertinent applications of symbiotic bioabsorbable devices within these therapeutic platforms.

Critical evaluations involve examining the systemic toxicity of chemical components on organs, assessing the carcinogenic potential in cancer cell development, evaluating the implantability within local tissues, ensuring blood compatibility, and studying the degradation of the resulting byproducts. In this section, we detail the application of bioabsorbable integration in biological diagnostics and treatment.

### 4.1 Diagnostic Platforms

#### 4.1.1 Brain Monitoring

Monitoring specific conditions of the body (such as the blood and brain) is valuable in modern medicine. This can be achieved through bioabsorbable devices. Monitoring the physiological signals of the brain is a key part of human health and recovery after surgery, and real‐time monitoring of brain physiological activities can be achieved by monitoring neural activity, intracranial pressure, temperature changes, and neurochemicals through bioabsorbable platforms.

**Figure 13:**
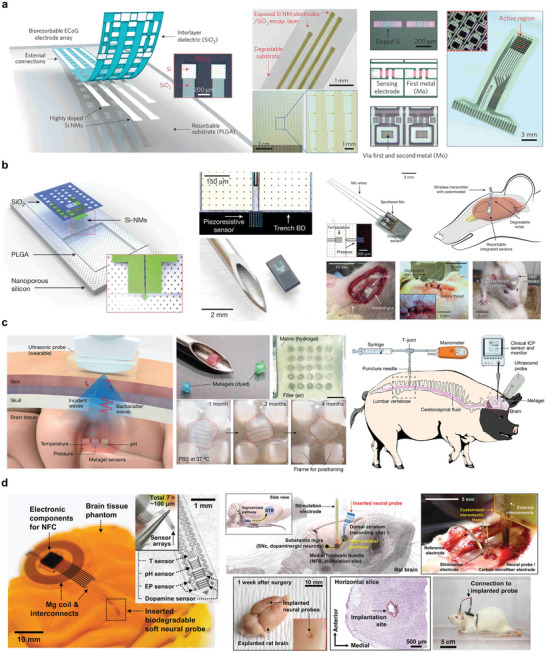
Brain monitoring. a) Schematic decomposition of neural electrode array structure and optical microscopic image. Reproduced with permission.^[^ ^30^ ^]^ Copyright 2016, Springer Nature Limited. b) Schematic diagram and in vivo experimental demonstration of biodegradable pressure sensor. Reproduced with permission.^[^ ^13^ ^]^ Copyright 2016, Springer Nature. c) Hydrogel diagram for wireless intracranial physiological sensor and in vivo experiment demonstration. Reproduced with permission.^[^ ^140^ ^]^ Copyright 2024, Springer Nature. d) Electronic sensor array of deep brain neurochemicals and schematic diagram of in vivo experiment. Reproduced with permission.^[^ ^55^ ^]^ Copyright 2022, Wiley‐VCH GmbH.

Si NMs are of great interest for monitoring brain physiological activity owing to their good electronic properties, mechanical flexibility, and ability to make contact with brain tissue in complex and temporal dynamics. One example is the synthesis of device‐level monocrystalline silicon nanomembranes with high‐resolution and high‐channel‐count neural interfaces. The interface used highly doped Si NMs to construct a neural interface electrode, Si NMs with a patterned doping region as the active semiconductor layer, and Mo as the electrode. Three layers of SiO_2_/Si_3_N_4_/SiO_2_ were used as the intermediate‐layer medium/package structure, and three layers of PLGA were used as the substrate (**Figure** **13**a). The spatial distribution of the evoked potential amplitudes measured on the surface of the rat cortex demonstrated the ability of the method to record continuously for several days at a high speed.^[^ ^30^ ^]^ Figure 13b illustrates a bioabsorbable brain‐monitoring silicon electronic sensor that combines PLGA with silicon nanomembranes to form a specific structure while covering it with a layer of silicon oxide that acts as an electrical insulation and fluid barrier. In the in vivo experiments on rats with sensors implanted in the brain, the monitoring data matched the data obtained from permanent sensors.^[^ ^13^ ^]^

Additionally, it is important to achieve real‐time assessment in line with the motor function recovery state window. One example is the preparation of oriented PLLA/BTO fiber films by electrospinning a dispersion of the PLLA solution of BTO NPs using polycaprolactone (PCL) as the encapsulation layer and degradable molybdenum (Mo) as the electrode layer to form a sandwich structure with the PLLA/BTO fiber film for PBPS. Through biological experiments in rats, it was found that PBPS could reach Vmax of 1.8 V and Imax of 30 nA. Simultaneously, as the nerve repair progressed, the signal output of the sensor gradually increased and showed a response performance similar to that of complete EMG.^[^ ^70^ ^]^ Hydrogels also play key roles in brain testing. Figure 13c presents an injectable ultrasonic sensor for the wireless monitoring of intracranial signals. A dual‐network hydrogel was prepared by combining PVA and chitosan. The gel is sensitive to PH and temperature. The gel reliably detected traumatic brain injury in rats over 24 d in vivo.^[^ ^140^ ^]^

Neurotransmitters are key substances that evaluate the physiological activity of the brain and the basic functions of the body and play a key role in regulating physiological signals. One example is a bioabsorbable silicon‐based neurochemical analyzer consisting of doped single silicon nanotubes as the current collector, 2D TMDs (MoS_2_ and WS_2_) as the active sensing electrode, Fe nanoparticles as the catalyst, Mg as the interconnect, SiO_2_ as the interlayer/insulator, and PLGA as the substrate and package (Figure 13d). The working principle is that because the dopamine molecule is positively charged, it can be combined with the negatively charged TMD‐Fe, and dopamine and TMD‐Fe oxidation to form a dopamine ketone, which causes changes in the electrical signal related to dopamine concentration, reflecting the dynamic change of dopamine concentration in the body.^[^ ^55^ ^]^

#### 4.1.2 Vascular Pressure and Blood Monitoring

**Figure 14:**
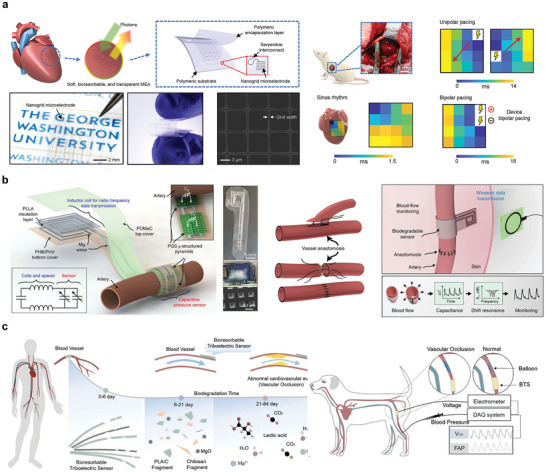
Vascular pressure and blood monitoring. a) MEA diagram and performance characterization for multi‐mode photoelectric heart monitoring. Reproduced with permission.^[^ ^141^ ^]^ Copyright 2023, The American Association for the Advancement of Science. b) Schematic diagram of double coil structure and vessel wrapping for wireless data transmission. Reproduced with permission.^[^ ^17^ ^]^ Copyright 2019, The Author(s), under exclusive licence to Springer Nature Limited. c) Structure and animal experimental demonstration of implantable bioabsorbable self‐powered sensor with triboelectric effect. Reproduced with permission.^[^ ^142^ ^]^ 2021 Wiley‐VCH GmbH.

Bioabsorbable integration is widely used for blood and cardiovascular monitoring. Transparent microelectrode arrays are important in the research and treatment of heart diseases. A flexible, fully bioabsorbable, and transparent microelectrode array (MEA) platform for bidirectional heart interfaces has been developed, utilizing PLGA as the substrate, electron beam lithography to create transparent Mo nanomaterials for the MEA layer, a lithographically interconnected layer, and a PLGA encapsulation layer (**Figure** **14**a). This platform was subsequently utilized for electrogram (EG) mapping on rat hearts and sections of human ventricular tissue, effectively demonstrating the capability of the Mo nanomaterial‐based MEA to interrogate three pivotal cardiac parameters without any hindrance.^[^ ^141^ ^]^

Based on the sensor device introduced in Section 3, a capacitive sensor composed of a double‐layer coil structure connected to an inductive structure composed of an edge field capacitor element was reported. The structure uses Mg as a monitoring platform to connect the PGS of the dielectric layer and the POMaC and PHB/PHV of the packaging layer (Figure 14b), as well as the PLLA separation material for the double‐layer coil structure. This was then connected to the upper‐pulse blood flow using a UV‐curable bioabsorbable sealant, which can cause a change in the vessel diameter, resulting in a capacitive response to a change in the LC oscillator resonant frequency. After the platform was implanted into the organism, capacitance changes could be monitored wirelessly by inductive coupling with an external coil. Similar results were obtained for wireless monitoring of arterial dilation, with measured pulse rates that closely matched the results of external Doppler ultrasound.^[^ ^17^ ^]^ One example is a pressure sensor composed of bioabsorbable materials with the triboelectric effect (Figure 14c). This device can successfully identify abnormal vascular occlusion events in large animals based on signal changes. The BTS has a service life of up to 5 d and a service efficiency of up to 5.95%.^[^ ^142^ ^]^

### 4.2 Tissue Regeneration

#### 4.2.1 Nerve Repair

Bioabsorbable electrical stimulation devices can be used in various forms of electrical stimulation therapy, and galvanic cells, the simplest battery devices, have been widely used in the field of electrical stimulation nerve repair.

**Figure 15:**
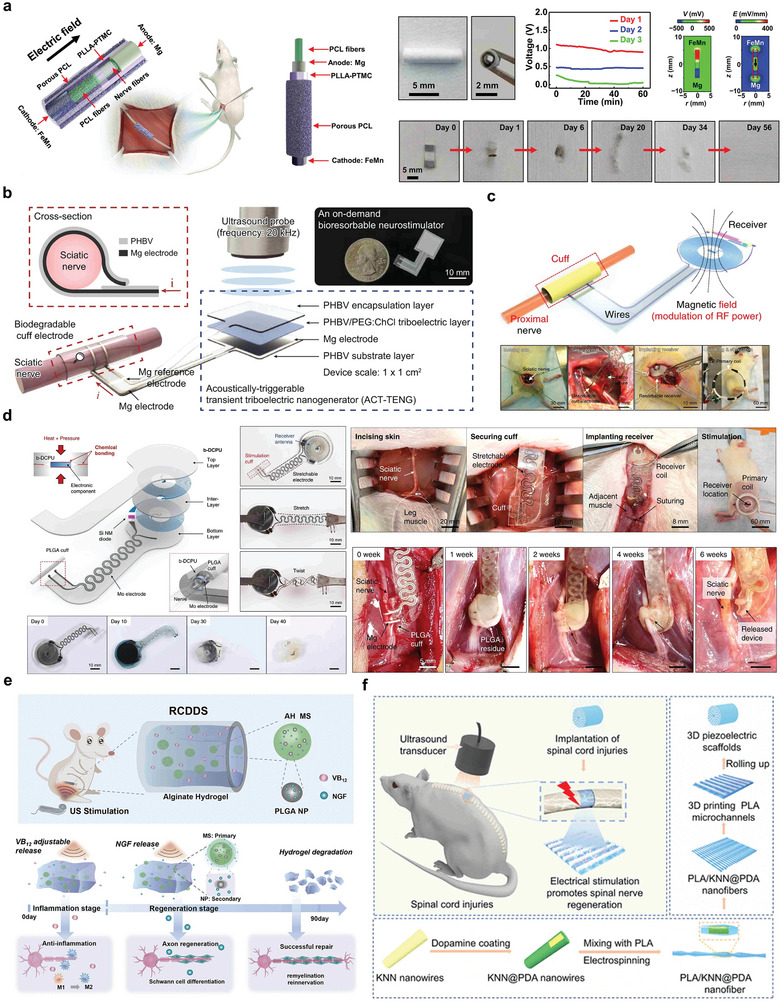
Nerve repair. a) Schematic illustration and explanation of the behavior of catheter‐like biodegradable and self‐powered neural repair devices. Reproduced with permission.^[^ ^143^ ^]^ Copyright 2020, The American Association for the Advancement of Science. b) Schematic diagram of ACT‐TENG with sleeve band and demonstration of nerve repair. Reproduced with permission.^[^ ^53^ ^]^ Copyright 2023, Springer Nature. c) Structure diagram of electrical stimulation device and effect of sciatic nerve implantation and repair. Reproduced with permission.^[^ ^144^ ^]^ Copyright 2018, Springer Nature America. d) Electrical stimulation device structure diagram and repair effect diagram of neural repair device. Reproduced with permission.^[^ ^146^ ^]^ Copyright 2020, Springer Nature. e) Schematic of the structure of RCDDS and the drug release process. Reproduced with permission.^[^ ^147^ ^]^ Copyright 2023, Royal Society of Chemistry. f) Schematic diagram and synthesis process of biodegradable 3D piezoelectric scaffolds promoting spinal cord injury repair by electrical stimulation. Reproduced with permission.^[^ ^38^ ^]^ Copyright 2022, American Chemical Society.

One study reported a biodegradable galvanic cell catheter device that could be used to promote peripheral nerve regeneration, consisting of a PLLA–PTMC membrane as the inner layer of the catheter and a porous PCL membrane as the outer layer of the catheter (**Figure** **15**a). By coupling the protocell and the neural guide catheter together, it has been demonstrated that sciatic nerve regeneration in rodents can be promoted by promoting the growth of nerve tissue and accelerating the recovery of motor function.^[^ ^143^ ^]^ Another example of electrical stimulation for nerve repair is the use of Mg electrodes and PHBV membranes to form cuff electrodes that can be wound around nerves to generate electrical stimulation. The triboelectric signal generated by the device flows through the Mg electrode lead of the cuff electrode to repair the injured nerves (Figure 15b). Low‐intensity ultrasound can effectively promote the physiological repair of injured sciatic nerves in the dermis in mouse model experiments.^[^ ^53^ ^]^

In addition to the biodegradable integrated galvanic cell structures that can be used for nerve repair, electrical stimulation can also be achieved using the wireless absorbable electronic systems described in Section 3. For example, Figure 15c shows a bioabsorbable electrical stimulation electronic system integrated with a radio‐frequency energy collector and an electrical interface with the peripheral nerve. The collector consisted of a double‐layer, double‐coil structure Mg ring antenna, a PLGA dielectric layer, a doped silicon nanomembrane RF diode, and a parallel plate capacitor with Mg electrodes. A Mg conducting plane was used above and below the silica dielectric layer. The electrical interface used a biodegradable metal strip (Mg/Mo) PLGA embedded in the receiver antenna to deliver electrical stimulation to the tissue. In biological experiments, radiofrequency electrical stimulation has been found to increase the axon regeneration rate during this critical stage of muscle recovery, reduce the time of muscle nerve regeneration, and improve the muscle mass of the tibialis anterior muscle and EDL.^[^ ^144^ ^]^

In addition to the electrical stimulation platform of the biodegradation mechanism, rapid elimination of the device through external stimulation after the integrated device has completed its operation is an important bioabsorbable direction. One example is an on‐demand bioabsorbable neurostimulator consisting of an acoustically triggered transient friction nanogenerator and a bioabsorbable cuff electrode, which is a bioabsorbable energy‐harvesting system that enables noninvasive elimination via a sound source. The bioabsorbable cuff electrode, consisting of a pair of Mg electrodes and a PHBV membrane, delivered the pulse to the target site of the sciatic nerve, and the ACT‐TENG produced stable electrical energy for 120 min at 0.5 W cm^−2^. In vivo experiments showed that the device was completely eliminated within 120 min in mice.^[^ ^145^ ^]^

Figure 15d reports a bioabsorbable electronic stimulator for promoting neuromuscular regeneration. The simulator uses b‐DCPU polymer as the base, Mg or Mo as the receiving antenna, and a PIN diode of SiO_2_ mono‐crystal film as the electrical stimulation device, which can generate biphase electrical pulses at low‐frequency signal (20 Hz) with RF power to stimulate nerve repair. In vivo experiments in mice, electrical stimulation proved that the device had a good promotion effect on neuromuscular repair, and had an enhanced effect on intraductal nerve regeneration at 6 weeks.^[^ ^146^ ^]^

Figure 15e shows a drug delivery stent that fits the treatment time window by constructing MS‐mixed hydrogels loaded with therapeutic drugs. Its main structure is an ultrasonically responsive alginate layered polymer network. The hydrogel network wraps vitamins B12 and NGF, which can select different treatment times for Windows according to specific conditions and flexibly adjust the release behavior of the two drugs through ultrasonic stimulation to achieve personalized treatment (Figure 15e). In vivo experiments showed that this ultrasound‐responsive drug delivery platform can accelerate nerve regeneration and enhance the secretion of nutrients associated with nerve regeneration.^[^ ^147^ ^]^

Bioabsorbable therapeutic devices can also be used to repair and regenerate central nervous system damage. Figure 15f shows a 3D scaffold with electrical stimulation properties obtained by electrospinning PLA and PDA. In a rat model of spinal cord injury, 20 min (100 kPa) of ultrasound stimulation for 8 consecutive weeks significantly improved the recovery of musculoskeletal function.^[^ ^38^ ^]^

#### 4.2.2 Bone Repair

**Figure 16:**
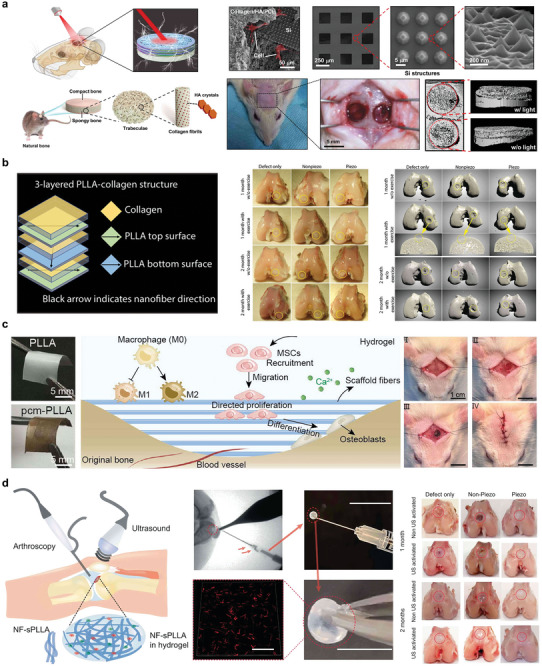
Bone repair. a) Schematic illustration of a silicon‐based 3D photoelectric scaffold in skull repair. Reproduced with permission.^[^ ^148^ ^]^ Copyright 2023, The American Association for the Advancement of Science. b) Structure diagram of piezoelectric PLLA scaffold and demonstration of bone repair effect. Reproduced with permission.^[^ ^149^ ^]^ Copyright 2022, The American Association for the Advancement of Science. c) Schematic diagram of PCM‐PLLA piezoelectric scaffold and experimental demonstration in vivo. Reproduced with permission.^[^ ^150^ ^]^ Copyright 2024, Science China Press. Published by Elsevier B.V. and Science China Press. All rights are reserved, including those for text and data mining, AI training, and similar technologies. d) Hydrogel bone repair diagram and animal bone repair effect diagram. Reproduced with permission.^[^ ^151^ ^]^ Copyright 2023, Springer Nature.

Bioabsorbable integrated platforms have a wide range of applications in bone repair. **Figure** **16**a introduces a 3D bionic photoelectric scaffold. The structure of the scaffold consisted of a porous matrix composed of hyaluronic acid nanocrystals mineralized by a collagen/PCL composite material to provide a microenvironment for tissue regeneration. Subsequently, a photoelectric network was formed by embedding a monocrystalline silicon film into a mineralized collagen/PCL scaffold. The network can further regulate the osteogenic differentiation of hBMSCs by generating an electrical signal that depolarizes the cell potential and induces intracellular calcium activity in response to near‐infrared illumination. In in vivo implantation experiments in rats, under near‐infrared irradiation, 4 weeks after implantation, the bottom of the defect cavity formed an obvious massive bone structure. The blank control group also showed almost no new bone formation 8 weeks after transplantation.^[^ ^148^ ^]^

Piezo‐effect bone repair scaffolds have also received considerable attention in the field of bone repair. For example, a biodegradable piezoelectric tissue scaffold is described in the Figure 16b, which achieves better piezoelectric and mechanical properties by constructing three PLLA scaffolds, each PLLA membrane is ≈15–25 µm thick, the collagen hydrogel layer is alternating between the layers, and each polylactic acid nanofiber film has an array of nanofibers. The fiber orientations of the first and third PLLA layers were parallel, and the middle layer was oriented at a 90° angle to the other two layers (Figure 16b). In the absence of exogenous TGF‐β, ES induced enhanced cartilage differentiation in stem cells and the introduction of voltage‐gated calcium channel inhibitors inhibited enhanced cartilage formation under piezoelectric stimulation.^[^ ^149^ ^]^

Another example of piezoelectric scaffolding that promotes bone regeneration is endogenous bone regeneration through a piezo‐induced controlled mineralized scaffold prepared by electrospinning a PLLA membrane immersed in a solution of dopamine hydrochloride. The PDA/PLLA was then immersed in an aqueous HA solution (Figure 16c). The PCM‐PLLA bracket exhibited the highest piezoelectric output power in the d_14_ direction, and its output voltage was ≈190 mV. Directional growth of scaffold fibers and their ability to release Ca^2+^ during degradation. Simultaneously, the piezoelectric properties of the scaffold were conducive to bone regeneration. Scaffolds facilitated the rapid recruitment of endogenous stem cells and promoted bone repair and regeneration in a rat skull model.^[^ ^150^ ^]^ Figure 16d shows an injectable and biodegradable piezoelectric hydrogel compounded by PLLA's cryogenic cutting of piezoelectric short nanofibers and a collagen matrix, which degrades into a completely safe by‐product after healing the damaged tissue. Biological experiments successfully guided cartilage differentiation, demonstrating the potential of this hydrogel for bone repair.^[^ ^151^ ^]^

#### 4.2.3 Wound Healing

**Figure 17:**
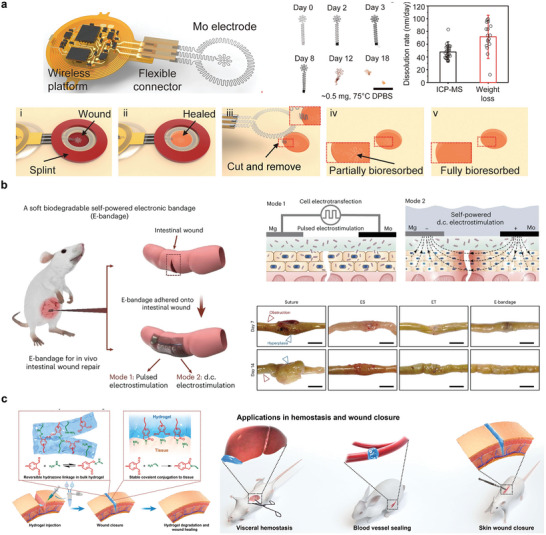
Wound Healing. a) Structure and degradation behavior of electrotherapy system for wound healing with electrical stimulation. Reproduced with permission.^[^ ^152^ ^]^ Copyright 2023, The American Association for the Advancement of Science. b) Structural diagram and in vivo experimental image of double electrostimulation electronic bandage. Reproduced with permission.^[^ ^153^ ^]^ Copyright 2024, Springer Nature. c) Structural diagram of HA‐PEG hydrogel and experimental demonstration of wound. Reproduced with permission.^[^ ^154^ ^]^ Copyright 2023, The American Association for the Advancement of Science.

Electrical stimulation therapy can ameliorate inflammation in chronic wounds. **Figure** **17**a shows a bioabsorbable electrical stimulation impedance sensing system consisting of Mo electrodes that achieves controllability of wound healing by externally connecting to a wireless platform. Subsequently, an experimental simulation is performed using a wound model. The electric‐field intensity near the inner electrode was ≈250 mV mm^−1^ (Figure 17a). The other areas of the fatty tissue accelerated the wound healing process; the wound healed faster than in the untreated group, with a healing rate of 86% in the treated group and 66% in the untreated group. Electrical stimulation completely closed the site after 18 d. The ability to combine these bioabsorbable power supply components suggests the possibility of a fully implantable, bioabsorbable wound‐healing system.^[^ ^152^ ^]^

Intestinal wound healing, which is a problem in traditional suture‐closure surgery and usually leads to long‐term difficulties in postoperative healing, can be accelerated using implantable biodegradable devices. A self‐powered electronic bandage based on double electrical stimulation can accelerate intestinal wound healing by sequentially sputtering thin layers of Mg and Mo onto a PCL substrate. In the innermost layer, a gel‐chitosan hydrogel layer that can be effectively attached to the intestinal tissue was used for direct contact. The treatment platform accelerated the healing of intestinal wounds through double electrical stimulation of the intestinal wounds (i.e., ET and ES) (Figure 17b). In vivo, tests showed that the microelectrode promoted protein exocytosis to maintain high activity within a week. In a specific intestinal wound model, e‐bandages provided a desirable degree of hyperplasia (at day 14, an improvement of ≈30%), whereas the e‐bandage group showed no significant intestinal obstruction. This is a promising approach for accelerating wound healing.^[^ ^153^ ^]^

Tissue adhesion has received attention from researchers as another option for post‐injury healing. Figure 17c shows a tissue adhesive and biodegradable hydrogel adhesive constructed using OPA/N‐nucleophile crosslinking HA and 4aPEG‐OPA. Coagulation was observed at 0.4 ± 0.1 min, and the coagulation time was 6.9 ± 1.4 min, which was significantly better than the commonly used fibrin gel. The potential application of the gel for hemostasis in vivo was demonstrated through implantation into animal models of liver and blood vessel injuries.^[^ ^154^ ^]^

### 4.3 Cardiac Pacing

Temporary cardiac pacing plays a crucial role in certain medical procedures, such as open‐heart surgery and in cases of cardiac disease. Traditional cardiac pacing devices typically consist of percutaneous leads, external wired power sources, and monitoring systems, which often require a second surgical procedure for removal after their function has been fulfilled. This not only poses an additional burden on the patient but also restricts their mobility during recovery. However, the use of bioabsorbable cardiac pacing offers a solution to these challenges.

**Figure 18:**
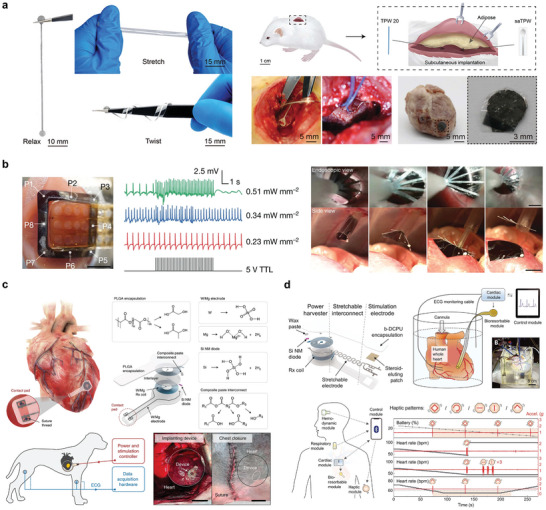
Cardiac pacing. a) Structure diagram and animal experimental image of bioabsorbable cardiac pacemaker lead. Reproduced with permission.^[^ ^155^ ^]^ 2021 Wiley‐VCH GmbH. b) Schematic diagram of monolithic silicon heart pacing with spatiotemporal light stimulation and experimental demonstration in animals. Reproduced with permission.^[^ ^156^ ^]^ Copyright, 2024 Springer Nature. c) Wireless bioabsorbable pacemaker structure and animal experimental images. Reproduced with permission.^[^ ^15^ ^]^ Copyright, 2021 Springer Nature. d) Schematic diagram and demonstration of transient closed‐loop system for temporary cardiac pacing. Reproduced with permission.^[^ ^157^ ^]^ Copyright 2022, The American Association for the Advancement of Science.

Bioabsorbable cardiac pacemaker leads are of great significance for wired cardiac pacing because they can degrade autonomously after use, avoiding mechanical damage during surgery and damage caused by a second operation to remove the lead. **Figure** **18**a shows a soft and absorbable temporary epicardial pacemaker wire composed of a degradable PLC and liquid metal, which maintains good electrical conductivity at strains exceeding 200%. In vivo experiments in SD rats and New Zealand rabbits showed that they could successfully pacify and degrade in animals without causing harm.^[^ ^155^ ^]^

A silicon‐based pacemaker with high temporal and spatial translational light stimulation utilizes four different photodiodes: single‐crystal p‐n Si, p‐i‐n Si, and p‐i‐n Si modified by Au nanoparticles realize the cathode process mainly through the diffusion of charge carriers, which produce more delocalized photochemical reactions owing to the higher diffusion length of charge carriers. The reliability of the pacing was demonstrated at a high frequency of 600 bpm. After using a 4 cm^−2^ Por‐Si instrument on the epicardial wall of the pig right ventricle, optical pacing was captured on biomedical ECG grid paper with different ventricular pacing waveforms (Figure 16b). At the same time, the device can be used in minimally invasive device delivery technology for optical heart modulation, requiring only a small incision (0.5–1 cm diameter) between the two ribs to realize the development of the Si membrane device and firmly placed on the surface of the epicardium.^[^ ^156^ ^]^

Another example involves the use of a pair of dissolvable metal electrodes integrated with contact pads attached to the myocardium for cardiac pacing. The wireless energy‐harvesting part of the system included a tungsten‐coated magnesium double‐layer dual‐coil loop antenna, a PLGA dielectric intermediate layer, and a radio frequency (RF) PIN diode based on doped monocrystalline silicon nanofilms (Si NM). The electrical extension and connector ends use an open double‐layer electrode strip, thereby delivering electrical stimulation from the antenna to the heart muscles (Figure 18c). The feasibility of this pacemaker was demonstrated by in vivo testing during open‐heart surgery in a canine model, in which the ECG recorded ventricular pacing patterns with clear pacemaker peaks and QRS complexes. The observed latency of 50–60 ms and the delta waves at the beginning of each QRS complex confirmed that the bioabsorbable pacemaker successfully provided epicardial stimulation for pacing.^[^ ^15^ ^]^

Cardiac pacing can also be performed using a bioabsorbable module composed of an RF power collector consisting of an induction receiver coil synthesized by molybdenum and a PIN silicon nanofilm diode, as well as a stimulation electrode of an integrated steroid‐eluding patch at the cardiac interface, which is interconnected by stretchable Mo (Figure 18d). It can eliminate the need for an external power source and ECG monitoring equipment. Related cardiac pacing experiments in rodents and dogs have shown sustained long‐term pacing and biocompatibility.^[^ ^157^ ^]^

### 4.4 Neuromodulation

The use of photoresponsive nanomaterials as interfaces to apply photothermal, photochemical, or photocapacitive effects to neurons or neural tissues to regulate neural activity is an important link in biotherapy. Neuromodulation through bioabsorbable platforms can avoid the risk of injury and infection caused by secondary surgery, and thus attract people's attention.

**Figure 19:**
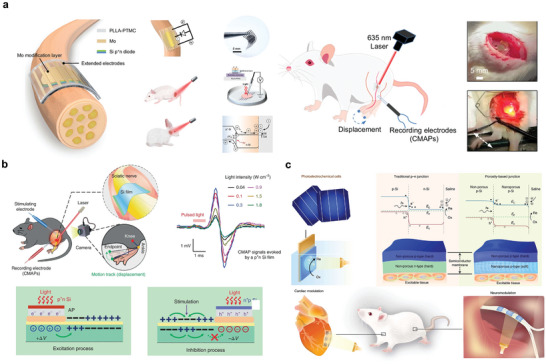
Neuromodulation. a) Optical image of biodegradable flexible photostimulated neural interface and schematic diagram of in vivo experiment. Reproduced with permission.^[^ ^158^ ^]^ Copyright, 2024 Springer Nature. b) Schematic diagram of p+n and n+p si membranes photostimulating and inhibiting peripheral nerve activity in vivo. Reproduced with permission.^[^ ^139^ ^]^ Copyright, 2022 Springer Nature. c) Photoelectric chemistry diagram of nano‐porous/non‐porous silicon materials and in vivo experimental demonstration. Reproduced with permission.^[^ ^159^ ^]^ Copyright, 2022 Springer Nature.

**Figure** **19**a shows a biodegradable flexible neural interface that can be used for peripheral nerve transdermal photoregulation. By p‐doping in n‐type silicon, p+ n thin‐film silicon diodes are prepared, and then a layer of Mo or Au metal is sputtered onto the thin film to ensure high excitation efficiency, charge injection, and sufficient light transparency. In vitro, photoelectric tests were performed on pure Si diodes (Si devices), Mo‐modified Si diodes, and Au‐modified Si diodes (Si/Au devices), and it was found that Mo‐modified devices exhibited the largest photovoltage, which was almost twice that of Si/Au devices.^[^ ^158^ ^]^

Another biodegradable neuroregulatory silicon‐based device is the generation of different types of PN junction silicon‐based devices by ion implantation and then defining the film pattern by photolithography (Figure 19b). In a rat model of dorsal root ganglion neurons, continuous 5 s irradiation of the p+Si membrane increased the membrane potential and led to cell depolarization, whereas on the n+Si membrane, the same conditions of illumination reduced the membrane potential and led to cell hyperpolarization. The APs were completely suppressed at ≈1.5W cm^−2^ for 60 s. The photosuppression effect induced by the device platform has a large hyperpolarization potential (≈10 mV), lasts for a long time (>60 s), and can be quickly recovered, which has not been previously reported. Thin‐film silicon diodes were applied to the peripheral nervous system to evaluate their photoexcitation and inhibition of sciatic nerves in vivo. The 635 nm light remotely incident on the Si film proved the existence of muscle complex action potentials (CMAPs) and displacement of the hind limb motion trajectory. Pulsed‐light irradiation of the p+ NSI film stimulated cmap, resulting in hind limb lifting. Photoinduced inhibition can be achieved alternately using an inverted structure (n+p silicon diode). The negative charge (−ΔV) generated by the light in the nerve can block the transmission from the proximal to the distal location, inhibiting cmap.^[^ ^139^ ^]^

In another study, nanoporous/non‐porous Si heterojunctions were created in P‐type Si, where nanoporous and microcolumnar Si heterojunctions were directly generated. In in vitro cardiac experiments, stimulating the heart with 532 and 808 nm lasers can successfully achieve effective depolarization of the myocardium (Figure 19c), with the heart immediately synchronized with the frequency of the light pulse, while 808 nm can penetrate deep tissue for optical bioregulation. In a rat model of acute sciatic nerve injury, the action potential of the lower limbs can be generated by irradiation with pulsed light.^[^ ^159^ ^]^

### 4.5 Drug Release

Traditional drug delivery methods involve an oral drug delivery system or local injection through a syringe, which has defects in the precise location of drug treatment and dosage control. An attractive alternative is the active control of drug release through bioabsorbable implantable drug delivery platforms, which offer precise treatment options.^[^ ^160^ ^]^ These drug release platforms can be categorized into two main types. The first type is a passive release platform, including bioabsorbable matrices and porous scaffolds that incorporate target drugs during synthesis and release them gradually over time through the degradation process, facilitating passive treatment. The second type is an active drug release platform, where implantable electronic devices, responsive to external thermal, acoustic, or electrical stimuli, regulate the degradation of the implant and thereby achieve precise control over drug release.

**Figure 20:**
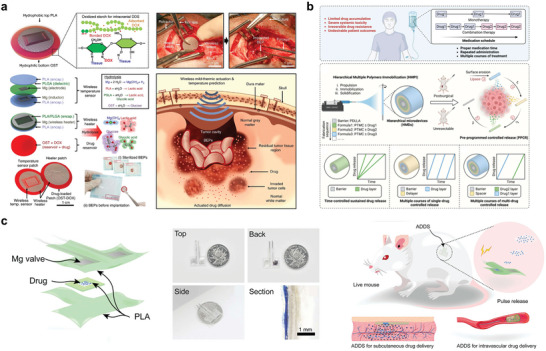
Drug release. a) BEP material design structure, drug diagram, and craniotomy image. Reproduced with permission.^[^ ^161^ ^]^ Copyright 2019, Springer Nature. b) Schematic diagram of HMPI controlled drug release design and application. Reproduced with permission.^[^ ^162^ ^]^ Copyright 2023, Wiley‐VCH GmbH. c) ADDS structure diagram and application demonstration. Reproduced with permission.^[^ ^163^ ^]^ Copyright 2023, Wiley‐VCH GmbH.

Heat‐responsive implantable drug‐delivery devices are simple and reliable solutions. **Figure** **20**a shows a biodegradable wireless device for drug delivery to brain tumors via thermal response, consisting of a hydrophilic drug‐filled OST film, in which devices are embedded as a microthermal drug delivery‐driven wireless heater and a controlled microthermal‐driven wireless temperature sensor. The device was constructed as a bioabsorbable electronic patch (BEP), which was externally applied to wirelessly drive the heater in the BEP through an alternating radio‐frequency magnetic field, releasing the drug from the repository, accelerating the diffusion of the drug between cells, and enhancing the depth of drug penetration. Craniotomy brain experiments in a canine GBM model showed that alternating radiofrequency magnetic fields allow long‐distance wireless energy to be transmitted through the tissue, which activates the warm drive that promotes the spread of the drug to tiny residual tumors that invade the normal brain tissue.^[^ ^161^ ^]^

Photoresponsive implant delivery systems are important. Figure 20b describes a method through the pre‐design of multiple drug‐carrying polymer formulations, highly controllable layered microdevices (HMD) with high resolution can be generated to achieve controlled release of embedded drugs (Figure 20b). The controlled release of drugs is mainly achieved through the controlled modification of the devices to form a controlled degradation rate in vivo. The antitumor effect of the device was evaluated using a mouse model of pancreatic tumor. The device successfully achieved multicourse‐controlled drug release, significantly inhibited pancreatic tumor recurrence, and exhibited excellent biosafety.^[^ ^162^ ^]^

Another example of controlled drug release through metal corrosion is the rapid release of drugs from drug reservoirs through magnesium deactivation control valves and long‐term delivery through the biodegradation of on‐demand drug delivery systems (DDS) (Figure 20c). The system was then implanted into the backs of rats to verify the pulsating release function of the system in vivo. After controlled release of NO into the body, the concentration of NO increased significantly to 10.3 µmol L^−1^.^[^ ^163^ ^]^

## 5 Conclusion and Outlook

**Figure 21:**
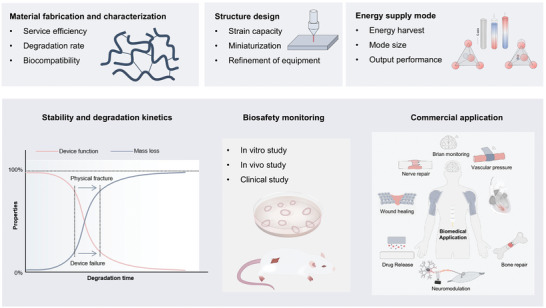
Future development roadmap of symbiotic bioabsorbable devices.

In this review, we explored the development and recent achievements of symbiotic implantable devices and their related therapeutic platforms, highlighting their unique advantages in biotherapy and their potential for future medical treatment. We focused on the degradation mechanism of symbiotic bioabsorbable materials, which determine the functional time of the device. We introduce different types of implantable devices and their integrated platforms in vivo, which play important roles in tissue repair, cardiac pacing, and physiological signal monitoring. In the roadmap (**Figure** **21**), we summarize future directions of symbiont absorbable devices and discuss the potential for future commercialization of symbiont absorbable electronic.

Service efficiency is an important factor restricting the commercialization of symbiotic bioabsorbable devices. Despite substantial progress in biodegradable materials, the service life of symbiotic bioabsorbable electronic devices (SBE) is still relatively short. Service efficiency is the ratio of the working life of SBE devices in vivo to the time to degrade in vivo. At present, the service efficiency of most SEB devices is less than 20%, which limits the wide application of SEB. The use of controlled degradation technology is expected to improve the service efficiency. When SEB completes the service work, the accelerated degradation of SEB is triggered by external physical factors such as light, heat, and ultrasound. This approach can effectively improve the efficiency of SEBs in service and lay the foundation for the development of more reliable, effective, and safe SEBs for personalized medicine. The second point is the controllability of the degradation rate. The different degradation mechanisms of different composition materials lead to the difference in the degradation time of SBE, and improve the long‐term stability and degradation control of the device to ensure that the device can maintain the expected mechanical properties and functions during the working cycle in the body. By precisely controlling the rate and way of material degradation, the device can gradually adapt to the changes in the tissue in the appropriate time, while avoiding excessive mechanical burden on the tissue. The third point is the development of more biocompatible materials, and future bioabsorbable electronics will use more advanced materials that not only have good biocompatibility, but also exhibit superior mechanical and electrical properties in the body. As the understanding of biological responses increases, bioabsorbable electronics will increasingly be designed in a personalized way. This includes tailoring the device to the specific biometric and pathological state of the patient to optimize its biocompatibility and safety. Personalized design can improve treatment effectiveness and reduce adverse reactions. Future studies will focus more on the long‐term biosafety of the device, conducting more comprehensive toxicity testing and long‐term efficacy evaluation. This includes an in‐depth analysis of the device's degradation products in the body to ensure that they do not cause toxic reactions or negative health effects.

In terms of device structure, integration and miniaturization are also problems that need to be solved for future commercialization. Miniaturization can improve the practicality and comfort of SEB and expand its clinical application field. Nanotechnology will play an important role in the miniaturization of SEBs. Nanomaterials and nano‐processing technologies can enable smaller and finer devices while maintaining or enhancing their functional performance. The development of high‐density integration miniaturization will be accompanied by higher functional integration. In order to guarantee the therapeutic effect while achieving the miniaturization of SEB, a number of possible directions should be considered, such as the design of novel structures for more compact layouts, the use of ultra‐thin packaging materials and methods, and the application of flexible integrated electronic circuits. Advances in biodegradable materials, electronics, micromachining, and nanomachining technology provide an excellent opportunity to develop smaller, more durable, and more powerful SEBs.

The power supply part of the device also restricts the future commercial application, the current energy supply methods for bioabsorbable electrical stimulation therapy are mainly divided into three categories: biological energy self‐drive, RF energy transmission, and galvanic battery. The biological self‐drive technology has the characteristics of no external energy supply and high output voltage, but the current is small, and the movement energy of limbs and organs needs to be transformed to provide electrical stimulation, which has specific requirements for the implant site, and the output is not stable enough to limit its application range and therapeutic effect. RF energy transmission technology has the characteristics of high voltage and current, and controllable electrical stimulation pulse frequency and width parameters. However, its transmission efficiency decayed rapidly with the depth of implantation, and the intensity of electrical stimulation was susceptible to the change of the spatial position between the stimulation end and the RF RF emission end. At the same time, the implantation end required a large area, which limited its application range and stability. Future devices will employ more efficient energy harvesting techniques, such as using bioelectrical signals, thermal energy, or mechanical energy in the body to power electronic devices. These technologies can improve energy harvesting efficiency by optimizing design and materials, ensuring that devices are self‐sufficient. Improve energy storage technology to achieve long life and efficient energy storage. This includes the development of novel biodegradable batteries and supercapacitors that can be adapted to the long‐term use needs of devices and safely degrade during final absorption.

Symbiotic implantable device is a new multi‐functional technology in implantable medical devices, which has important application value in future medical applications. By continuing to develop symbiotic bioabsorbable materials, explore new structures for symbiotic biodevices, and promote the development of relevant therapeutic platforms to develop more effective, biosafe, and human tissue‐compatible materials to improve patient outcomes and quality of life. By developing symbiotic bioabsorbable materials, personalized design of devices, improving the output efficiency of energy components, and more comprehensive verification of the biosafety of devices, symbiotic bioabsorbable electronic devices will become an important part of future healthcare.

## Conflict of Interest

The authors declare no conflict of interest.
